# Perturbation-based balance training on treadmills for falls prevention in older adults: a review of training protocols and reporting recommendations (ProRePBT)

**DOI:** 10.1186/s12877-026-07124-3

**Published:** 2026-02-14

**Authors:** Nina Marie Schmidt, Pia Ruess, Tobias Morat, Christopher McCrum, Christian Werner, Michael Schwenk, Tim Fleiner

**Affiliations:** 1 Department for Health Services Research, Geriatric Medicine, School of Medicine and Health Services, Carl von Ossietzky University Oldenburg, Oldenburg, Germany; 2 Department of Human Movement, Training and Active Aging, Institute of Sports and Sports Sciences, Heidelberg University, Heidelberg, Germany; 3 Institute of Movement and Sport Gerontology, German Sport University Cologne, Cologne, Germany; 4 Department of Nutrition and Movement Sciences, NUTRIM School of Nutrition and Translational Research in Metabolism, Maastricht University Medical Centre+, Maastricht, The Netherlands; 5 Geriatric Center, Medical Faculty Heidelberg, Heidelberg University, Heidelberg, Germany; 6 Department of Sport Science, Human Performance Research Center, University of Konstanz, Constance, Germany; 7 Institute for Geriatric Research, Ulm University Medical Center, Ulm, Germany; 8 Institute for Medical Engineering and Mechatronics, Ulm University of Applied Sciences, Ulm, Germany

**Keywords:** Falls prevention, Perturbation training, Treadmill, Reactive balance, Aging

## Abstract

**Background:**

Falls are a leading cause of injuries in older adults, often resulting from slipping and tripping. Perturbation-based balance training (PBT) applied on treadmills is an emerging task-specific approach to falls prevention but standardized training protocols are lacking. This review presents an overview of PBT protocols on treadmills, their theoretical justification and proposes recommendations for standardized reporting.

**Methods:**

A systematic search was conducted in PubMed, Embase, Web of Science, CINAHL, CENTRAL and Clinical Trials Registration on May 9, 2025 to identify RCTs, pilot studies, study protocols and trial registrations on treadmill-based PBT for falls prevention in healthy older adults or those diagnosed with stroke, Parkinson’s disease or multiple sclerosis. Studies were screened by two independent researchers. Data on general and PBT specific training parameters as well as the justification for those parameters were narratively synthesized.

**Results:**

1253 studies were identified. The eligibility criteria were met by 69 publications referring to 36 research projects. In total, 1928 participants were included, with 950 participants in the PBT groups. The training periods lasted from a single session to 12 weeks, including one to three sessions per week, each from 20 to 60 min. During standing and walking, from 24 up to 160 unannounced perturbations were induced, consisting of treadmill belt acceleration and deceleration as well as lateral displacements. Training intensity was often individually adjusted based on participant performance or subjective feedback, though methods varied widely. Perturbation frequency timing and the perturbed leg, as well as the theoretical justification for the training parameters were rarely reported.

**Conclusions:**

The results reveal a high heterogeneity in the PBT protocols. Furthermore, training parameters and their justification were insufficiently reported in many studies. Therefore, we propose a reporting standard for PBT protocols (ProRePBT) to increase comparability between studies, improve replicability, and facilitate implementation into clinical practice.

**Supplementary Information:**

The online version contains supplementary material available at 10.1186/s12877-026-07124-3.

## Introduction

The global prevalence of falls in older adults is 26.5% [1]. According to the World Health Organization, falls are the second most common cause of unintentional injuries worldwide [2]. Adults over 60 are particularly affected, experiencing the highest number of falls [3]. Individuals with neurological diseases such as Parkinson's disease [4], multiple sclerosis [5], and stroke [6] are at an even greater risk [7]. Falls can have serious consequences, e.g. fractures, head injuries, fear of falls and increased dependency [8, 9]. Additionally, falls and their consequences can significantly impact the healthcare system [10, 11].

Exercise interventions can be effective for preventing falls in older adults [12]. Since falls frequently occur due to tripping and slipping while walking [13, 14], exercise that specifically targets the skills needed to cope with these situations should be considered [15, 16]. Perturbation-based balance training (PBT) is one task-specific approach to falls prevention, defined as "balance training that uses repeated, externally applied mechanical perturbations to trigger rapid reactions to regain postural stability in a safe and controlled environment" [17]. Despite the various approaches to this training method, studies have shown its high potential for task-specific falls prevention [18], primarily by training reactive neuromuscular responses that are critical for recovering balance after unexpected disturbances. However, several systematic reviews on the effectiveness of PBT highlight the lack of detailed information about the applied training protocols [18–20], making it difficult to understand which specific components contribute to the observed effects.

The perturbation treadmill is an increasingly used type of device for applying PBT that simulates the unexpected nature of falls in everyday life by inducing unannounced balance disturbances through acceleration and deceleration of the treadmill belt, mediolateral displacement, and sudden starts or stops [17]. This approach offers potential advantages over other PBT methods because perturbations are less predictable than in overground setups where the perturbations appear at a fixed location or in a consistent manner [21] and because treadmills require less space than overground setups in clinical settings [17].

The characteristics of PBT protocols have been the subject of previous discussions in relation to overground setups [21] and clinical settings [22]. However, treadmill-based PBT has become much more common and more training protocols and studies have been published, that are based on different theoretical foundations. Despite this progress, there is currently a limited evidence available regarding the underlying mechanisms of PBT [21] and how these are affected by different aspects of training dosage, such as the number of perturbations or their intensity and frequency. Moreover, there is currently no consensus on which training parameters are critical for PBT or how they should be reported. While established tools for reporting exercise interventions already exist, such as the Template for Intervention Description and Replication (TIDieR) checklist [23] or the Consensus on Exercise Reporting Template (CERT) [24], these frameworks primarily address general elements like session number, duration, intensity, and setting. Due to the task specific and reactive nature of PBT, more tailored reporting appears necessary. Since PBT aims to train reactive rather than proactive balance responses [17], specific parameters that are rarely captured by conventional reporting schemes, such as the predictability of perturbations, perturbation magnitude, direction and velocity, and the specific gait phase during which perturbations occur, are particularly relevant to report. Additionally, treadmill-specific factors including walking speed may substantially influence the training challenge and the underlying adaptation mechanisms.

Therefore, the aim of this review is to summarize the approaches and provide an overview of currently available treadmill PBT protocols for the prevention of falls in older adults. Specifically, we intend to map the chosen training characteristics and examine their theoretical justifications. Based on these findings, we propose key components for a standardized reporting checklist (ProRePBT) to be used in future studies, thereby enhancing transparency, comparability, and scientific progress in the field of PBT.

## Methods

The structured literature search was conducted on May 9, 2025, in the following databases: PubMed, Embase, Web of Science, CINAHL and CENTRAL. Additionally, the Clinical Trials Registration database was searched, as only information on the trial methods was relevant to our research objective. In the case of study registrations, the study register ID was utilized to conduct a search for corresponding published full texts that may provide further information on the intervention methods. The search syntax and eligibility criteria were based on the adapted population, intervention, comparison, outcome (PICO) scheme. In Table 1, the key search terms are shown. The syntax was adjusted individually for each database. The detailed search strategies can be found in Additional file 1.

**Table 1:** The key search terms based on the PICO scheme were used in combination with the operators “OR” and “AND”

PICO component	Key search terms
Population	Old adults; older; elderl*; senior*; aged; ageing; aging; stroke; apoplexy; cerebrovascular accident; brain infarction; brain ischemia; brain attack; cerebral infarction; intracranial hemorrhage; parkinson*; PD; multiple sclerosis; MS
Intervention	Perturbation; PBBT; PBT; slip; trip; reactive balance; treadmill
Control	Not applicable
Outcome	Fall*; balance; reactive recovery response; postural control; stability; gait; mobility; physical capacity; functional capacity; physical functioning

We included intervention studies (randomized controlled trials (RCTs), clinical studies), study protocols, working papers, preprints and trial registrations of RCTs and clinical studies. Healthy older adults and those diagnosed with stroke, Parkinson’s disease or multiple sclerosis were included because PBT was often used in these conditions. The mean age of the study population had to be at least 60 years. The intervention had to be focused on falls prevention training on a treadmill (single or split belt), where the perturbation was induced by the treadmill itself, often described as surface perturbation [25]. Only studies published in English or German were included. There was no restriction on the year of publication. Non-interventional and cross-sectional studies, as well as any type of review or meta-analysis were excluded. Further exclusion criteria were interventions in which the PBT was not applied by or on a treadmill, also including perturbations induced by the placement of objects on the treadmill belt.

### Study selection

All steps of the screening process were carried out by two independent reviewers (NMS and PR). Any disagreements were resolved by consensus through discussion. First, the studies were imported into Mendeley Reference Manager Version 2.118.0 (Elsevier Ltd., Amsterdam, Netherlands), and duplicates were removed. The titles and abstracts of the remaining articles were then screened for eligibility using Rayyan software [26]. Potentially relevant full records were then screened according to the eligibility criteria described above.

### Data extraction

Authors, year of publication, study design and general data on the study population (number of participants, mean age, sex, medical condition) were obtained. Information on the following training parameters was extracted and narratively synthesized: (1) type of treadmill; (2) training period and number of training sessions; (3) duration of total training session and duration of perturbation training; (4) type of perturbation; (5) predictability of perturbations (announcement of perturbations, randomization of perturbation types); (6) training intensity and progression; (7) frequency of perturbations; (8) number of perturbations per training session; (9) treadmill belt speed (walking perturbations); (10) gait event during perturbation (walking perturbations) and (11) perturbed leg (walking perturbations). Where possible, the justification for the training parameters was also extracted to gain a better understanding of how the training protocol features were chosen. The justification of a particular parameter selection was identified upon the explicit articulation of the objective underlying its choice, whether in the context of a theoretical concept or driven by pragmatic or feasibility considerations. Citation of prior work or the author's own experience were also considered as a basis for the argument. Additionally, due to the high heterogeneity in the definition of perturbations, in this paper, for consistency, perturbation types are described in terms of what the treadmill itself (e.g. displacement) or the treadmill belt (e.g. acceleration and deceleration) does. When perturbations of this type are induced while the person is standing on the treadmill, they are referred to as standing perturbations. Consequently, perturbations that occur during ambulation on the treadmill are termed "walking perturbations".

## Results

### Systematic search results

**Fig. 1:**
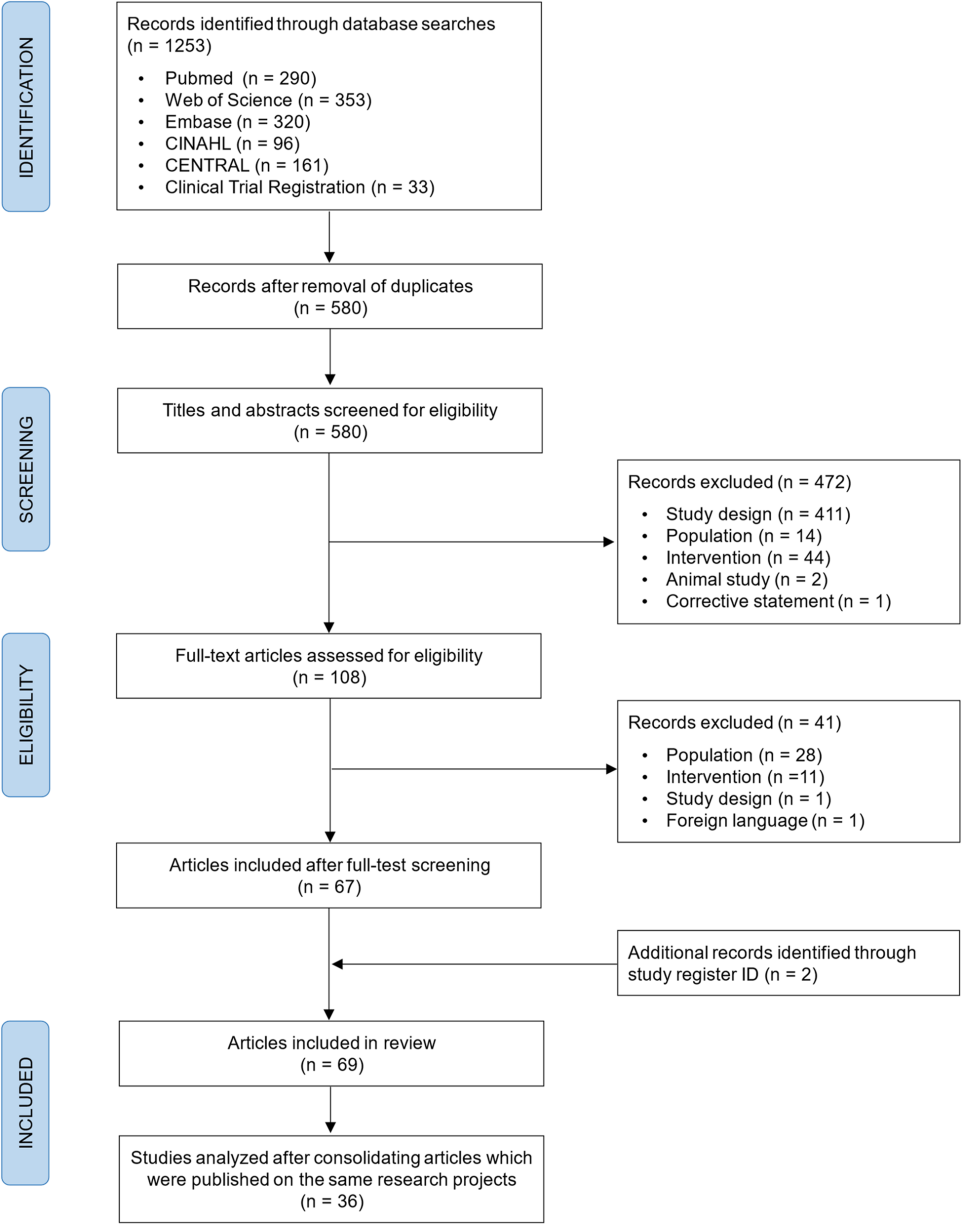
Flowchart of the systematic literature search and screening process for eligibility

The full screening process and reasons for exclusion are illustrated in Fig. 1. The database searches identified 1253 articles in total. After removing duplicates, the titles and abstracts of 580 publications were screened for eligibility. 108 full texts were assessed, and 67 articles met all the inclusion criteria. In addition, two eligible publications were identified via study register searches using the study registration ID, resulting in a total of 69 included articles. Several included articles originated from the same research projects (hereafter referred to as 'studies'). Consequently, these were consolidated for analysis by citing one article as the main study, while extracting data from all related publications. Related articles were identified based on the same study registration ID, sample characteristic and methods, and/or author team. The following section details a total of 36 different studies.

### Summary of included studies

The selected studies comprised 16 RCTs [25, 27–41], four two-group studies (two with a pretest–posttest design [42, 43], one longitudinal study [44] and one two-arm prospective controlled trial [45]), three single-group intervention studies [46–48], four pilot studies (three with a control group [49–51] and one without [52]), one pre-print for a pilot RCT [53], two study protocols (one for an RCT [54] and one for a pilot-controlled trial [55]), and six trial registrations (three for RCTs [56–58], two for three-group interventions [59, 60], and one for a two-group longitudinal study [61]). The publication period ranged from 2004 to 2025, with the majority of studies published in 2019 or later. In total, there were 1928 participants included in the eligible completed studies (1068 women). In addition, a total of 86 participants are planned in the two study protocols [54, 55]. Out of the six trial registrations, five reported an estimated enrollment totaling 562 participants [56–60], while one did not report a planned sample size [61]. The PBT groups accounted for a total of 950 participants in the studies conducted. An additional 61 participants were planned to receive the PBT intervention in the two study protocols [54, 55]. The trial registrations did not specify how many participants would be allocated to the PBT groups. The study sample consisted of healthy older adults in 17 studies [30, 33, 34, 37, 38, 40–44, 46, 49, 53, 54, 58, 60, 62] and fall-prone older adults or those who had previously suffered a fall in eight studies [25, 27, 29, 35, 39, 51, 55, 61]. In terms of other conditions, five studies included participants after a stroke [31, 47, 50, 52, 59], two included individuals with Parkinson’s disease [28, 36], two involved participants with cognitive impairment [56, 57], one involved people with degenerative cervical myelopathy [45], and one study did not further specify the participants' characteristics [48].

Eighteen studies recruited community-dwelling older adults [27, 29, 33, 34, 37, 38, 40, 42–44, 46, 47, 53–55, 58, 62], one of which focused on individuals living in rural communities [48]. Additionally, three studies recruited from senior housing facilities [30, 39, 49], one from a day-care facility [52], and two studies included hospitalized participants [31, 35]. Two studies recruited through physiotherapy practices [25, 51]. Three studies recruited participants following a clinic visit—one from a movement disorder clinic [28], one after a stay in a neurosurgical hospital [45], and one from a Parkinson’s disease research center [36]. In seven studies, the recruitment setting was not specified [41, 50, 56, 57, 59–61]. The average age of the participants in the PBT groups was 71.6 ± 6.3 years and for the control groups 72.6 ± 6.6 years – excluding the study from Lee et al. [62], which reported a mean age of 74.5 ± 6.9 years for both groups combined. Four studies even reported a mean age of over 80 years [35, 39, 49, 51]. One study only specified that participants were older than 60 years [48]. The study protocols plan to include participants older than 65 years [55] and older than 70 years [54]. Regarding the trial registrations, three aim to recruit participants over 65 years [56, 58, 61], one over 70 years [57], one between 65 and 80 years [60], and one between 56 and 90 years for healthy older adults and up to 89 years for participants with stroke [59]. The characteristics of the included studies are presented in Table 2.

**Table 2:** Characteristics of studies included in the analysis

Study	Study design		Population receiving PBT	
Allin et al., 2020 [42] (Madigan, 2018 [63])	2-group, pretest–posttest design		*n* = 11^a^, 5♀, 71.1 ± 3.3y. Community-dwelling, healthy	
Aviles et al., 2019 [49] (Aviles et al., 2020a [64]; Aviles et al., 2020b [65]; Madigan, 2015 [66])	Pilot controlled study		*n* = 19^a^, 13♀, 80.9 ± 6.2y. Independent residents of senior housing facilities, healthy	
Bhatt et al., 2018 [59]	Study protocol for pilot study (trial registration)		*n* = 90, INT1: 56-99y, healthy older adults; INT2: 18-90y, at least 6 months post-stroke	
Brüll et al., 2023 [27] (Hezel et al., 2024 [67]; Brüll, 2019a [68]; Brüll,2019b [69])	RCT		*n* = 23^a^, 13♀, 76.5 ± 5.3y. Community-dwelling or assisted living, fall prone (fall in the past 12 months or 8-level balance scale < 5)	
Cheng et al., 2020 [45]	2-arm prospective controlled clinical trial		*n* = 15^a^, 5♀, 64.0 ± 5.3y. Patients with degenerative cervical myelopathy that underwent a cervical decompression surgery	
Chien et al., 2018 [46]	Single group intervention		*n* = 17, 12♀, 68.3 ± 5.8y. Community-dwelling, healthy, recruited via advertisement in sports center	
Dusane et al., 2021 [47]	Single group intervention		*n* = 11, 2♀, 63.27 ± 8.2y. Community-dwelling, at least 6 months post-stroke	
Faria et al., 2023 [61]	2-group longitudinal study (trial registration)		INT1 (PBT in distributed manner): *n* = 20; INT2 (PBT on massive basis): *n* = 20, all participants ≥ 65y. Fall prone (≥ 1 fall in the past six months)	
Gassner et al., 2019 [28] (Steib et al., 2019 [70]; Klamroth et al., 2019 [71]; Steib et al., 2017 [72]; Gassner et al., 2017 [73]; Klamroth et al., 2016 [74])	RCT		*n* = 18^a^, 11♀, 67.6 ± 8.2y. People with Parkinson’s disease (Hoehn & Yahr disease stage mean = 2.6 ± 0.5)	
Gerards et al., 2023 [29]	RCT		*n* = 39^a^, 31♀, median = 73, IQR = 8y. Community-dwelling, fall in previous three months	
Gimmon et al., 2018 [30] (Kurz et al., 2016 [75]; Ronen, 2011 [76])	RCT		*n* = 21^a^, 13♀, 78.2 ± 5.6y. Protected housing residents, healthy	
Grabiner et al., 2012 [43]	2-group, pretest–posttest design		*n* = 22^a^, 22♀, 65.9 ± 7.8y. Community-dwelling women, healthy	
Handelzalts et al., 2019 [31] (Soroker, 2015 [77])	RCT		*n* = 16^a^, 4♀, 62.5 ± 8.4y. Hospitalized, unilateral stroke	
Hezel et al., 2023 [55] (Hezel et al., 2022 [78])	Study protocol for pilot controlled study		*n* = 36^a^, ≥ 65y. Community-dwelling, fall prone (Timed Up and Go > 12 s, habitual gait speed < 1.0 m/s and/or fall in the past 12 months)	
Lanza et al., 2024 [50] (Gray, 2014 [79])	Pilot controlled study		*n* = 18^a^, 4♀, 62.5 ± 7.1y. More than six months post-stroke with hemiparesis	
Lee et al., 2018 [62] (Lee et al., 2020 [32])	RCT		INT1 (24 belt decelerations): *n* = 15; INT2 (40 belt decelerations): n = 15, all participants 74.5 ± 6.9y, 34♀. Community-dwelling, healthy	
Liu et al., 2021 [33]	RCT		*n* = 53^a^, 36♀, 72.9 ± 6.6y. Community-dwelling, healthy	
Lurie et al., 2013 [51] (Lurie, 2009a [80]; Lurie, 2009b [81])	Pilot controlled study		*n* = 26^a^, 13♀, 81.1 ± 6.5y. Referred for gait/balance physical therapy, fall prone (considered for fall risk by their primary care provider)	
Lurie et al., 2020 [25]	RCT		*n* = 253^a^, 119♀, 78y. Referred for gait/balance physical therapy, fall prone (fall in past 12 months, Timed Up and Go > 13.5 s; or Dynamic Gait Index ≤ 19; or Berg Balance Scale < 50; or Activities-specific Balance Confidence < 67%)	
Montana State University, 2021 [58]	RCT		*n* = 16, ≥ 65y. Community-dwelling, healthy	
Nachmani et al., 2021 [54] (Shelef, 2017 [82])	Study protocol for RCT		All participants: *n = 50*, ≥ 70y. Community-dwelling, healthy	
Nørgaard et al., 2023 [34] (Nørgaard et al., 2024 [83]; Nørgaard et al., 2022 [84]; Nørgaard et al., 2021a [85]; Nørgaard et al., 2021b [86])	RCT		*n* = 70^a^, 41♀, 72.7 ± 4.7y. Community-dwelling, healthy	
Petrovic et al., 2024 [35] (Trampisch et al., 2023 [87])	RCT		*n* = 67^a^, 44♀, 80 ± 5y. Hospitalized, fall prone (fall in past 12 months)	
Protas et al., 2005 [36]	RCT		*n* = 9^a^, all men, 71.3 ± 7.4y. Diagnosed with idiopathic Parkinson’s Disease	
Punt et al., 2019 [52]	Pilot study		*n* = 10^a^, 3♀, 61.5 ± 7.2y. Patients in day-care centers for older adults, at least 21 months post-stroke, fall in previous six months	
Rieger et al., 2020 [37]	RCT		*n* = 15^a^, 8♀, 70.3 ± 4.0y. Community-dwelling, healthy	
Rieger et al., 2024 [38] (Vrije Universiteit, 2022 [88]; Rieger et al., 2020 [89]; Vrije Universiteit, 2019 [90])	RCT		*n* = 35^a^, 25♀, 75.5 ± 5.4y. Community-dwelling, healthy	
Shimada et al., 2004 [39]	RCT		*n* = 18^a^, 14♀, 81.8 ± 5.9y. Residents or outpatients of geriatric health service facility, muscle weakness and decreased balance and gait functions, fall prone (muscle weakness, decreased balance and gait functions; not further defined)	
US Department of Veterans Affairs, 2008 [41]	RCT		*n* = 27, 2♀, 73.0 ± 4.2y. Healthy	
Van Wouwe et al., 2021 [44]	2-group longitudinal study		*n* = 14^a^, 7♀, 70.8 ± 4.6y. Community-dwelling, healthy	
Virginia Polytechnic Institute and State University, 2022 [60]	3-group longitudinal study (trial registration)		*n* = 30, 65-85y. Healthy	
Wang et al., 2022 [40] (Wang et al., 2019 [91]; Bhatt, 2014a [92]; Bhatt 2014b [93])	RCT		*n* = 73^a^, 45♀, 72.5 ± 6.2y. Community-dwelling, healthy	
Whitten et al., 2023 [48]	Single group intervention (only abstract available)		*n* = 19, ≥ 60y. Community-dwelling in rural area	
Yang et al., 2021 [56]	RCT (trial registration)		*n* = 30, 65-90y. Mild Alzheimer’s Disease (Montreal Cognitive Assessment score 11–21 or Mini Mental Status Examination score 18–23)	
Zhu et al., 2025 [53]	Pilot controlled trial (pre-print)		*n* = 10^a^, 5♀, 67.1 ± 2.8y. Community-dwelling, healthy	
Zieschang, 2024a [57] (Zieschang, 2024b [94])	Study protocol for RCT (trial registration)		*n* = 396, ≥ 70y. Geriatric patients with and without cognitive impairment, fall prone (≥ 40% prospective fall risk and being capable of walking ≥ 70 m in a 2-Minute Walk)	

If the literature search revealed more than one publication on the same intervention study, the publication reporting the results was listed first. The related study protocol and/or clinical trial registration are given in parentheses. If only a study protocol was published, the inclusion criteria for participant age and the planned sample size are reported here

*RCT* randomized controlled trial, ♀ female; *y* years, *INT* intervention, *IQR* interquartile range, *PBT* Perturbation-based balance training

^a^Only treadmill perturbation training group (not including control groups)

### Perturbation treadmill

A comprehensive overview of the training parameters is presented in Additional file 2. Eight studies used the ActiveStep treadmill (Simbex, Lebanon, NH, United States) [25, 33, 40, 43, 47, 50, 51, 62], one study used the C-Mill VR + (Motek Medical B.V., Houten, The Netherlands) [38], one study used a custom-build treadmill [41] and four studies used regular treadmills [42, 49, 53, 61] capable of belt acceleration and deceleration perturbations. Four studies used the BalanceTutor™ (MediTouch LTD, Netanya, Israel), which includes, additional to belt acceleration and deceleration, lateral shifts that deliver mediolateral perturbations, causing participants to be displaced sideways from their balance [27, 31, 35, 55]. One study used the QQ-Mill (Motekforce Link, Amsterdam, The Netherlands), which can produce similar perturbations [45]. Two studies used the Balance Measure and Perturbation System (BaMPer System [95]) to apply both mediolateral and anteroposterior shifts without using belt acceleration or deceleration [30, 54]. Additionally, two studies used the Gait Real-time Analysis Interactive Lab (GRAIL; Motek Medical B.V., Houten, The Netherlands), which allows for platform pitching [37, 52], and two studies used the Computer Assisted Rehabilitation Environment (CAREN; Motek Medical B.V., Houten, The Netherlands), capable of producing platform translations and rotations in six degrees of freedom [29, 44]. One study used the Mercury treadmill (h/p/cosmos Sports & Medical GmbH, Nussdorf, Germany) mounted on a tiltable platform (Zebris Medical GmbH, Isny, Germany) to deliver three-dimensional tilting perturbations [28]. Three studies used split-belt treadmills [34, 39, 46], and seven studies did not report which type of perturbation treadmill was used [36, 48, 56–60].

### Number of training sessions and training period

**Fig. 2:**
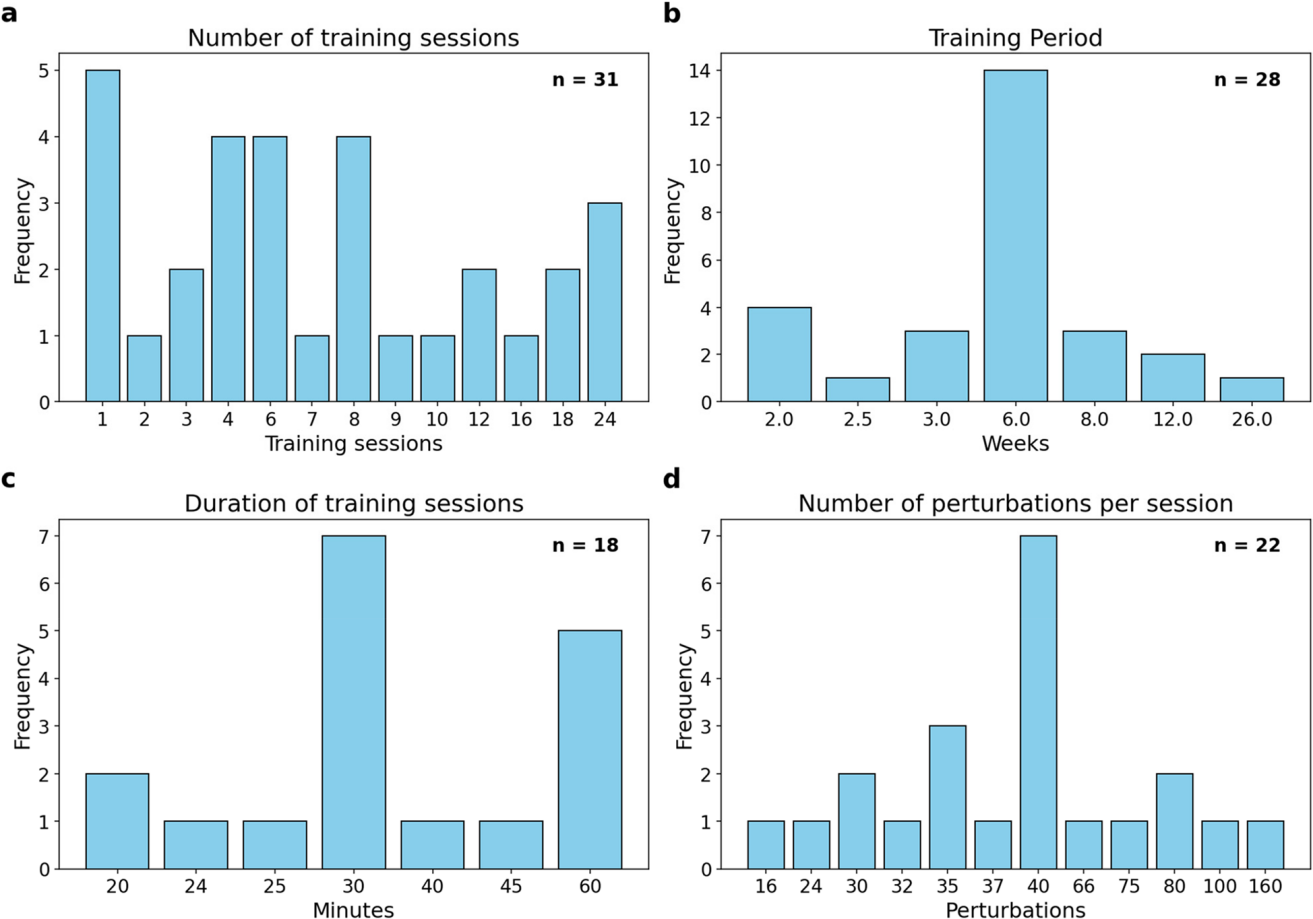
Variation in key training parameters across included PBT studies. Bar charts illustrating the heterogeneity of four numerically reported training parameters across included studies: (**a**) number of training sessions, (**b**) training period (weeks), (**c**) duration of each training session (minutes), and (**d**) number of perturbations per session.

Figure 2a and 2b provides a graphical overview of the number of training sessions and training period. Five studies included only one perturbation training session [33, 40, 53, 56, 62]. Eight studies included between two and four sessions [29, 35, 37, 42, 47, 55, 59, 61], 13 studies provided between six and 12 sessions [28, 31, 38, 41, 44, 45, 48, 49, 52, 54, 55, 57, 60], and six studies included between 16 and 24 sessions [27, 30, 36, 46, 50, 58]. Three other studies did not provide a specific number of sessions but rather a wide range [25, 43, 51]. The earliest RCT, published in 2004, did not specify the number of sessions but aimed for a total training time of 600 min [39].

In 22 studies, the training interventions were conducted over a period of two to six weeks [25, 27, 29, 31, 35, 37, 38, 41–45, 47–50, 52, 54, 55, 59–61]. Five studies spanned eight to twelve weeks [28, 30, 36, 46, 58], and one study extended over six months [39].

Training frequency was typically one to three sessions per week. However, two studies applied the training once daily [31, 59], and one study conducted sessions twice daily [61].

One study used a vaccination-like training protocol consisting of four sessions: two initial sessions on the same day, one in week three, and a 'booster session' in week 26 [34]. Two studies did not specify the frequency or period of the sessions [51, 57].

### Duration of training sessions

The duration of training sessions is visualized in Fig. 2c. Training sessions in total lasted between 20 and 30 min in 11 studies [27, 29–31, 33, 34, 40, 49, 54, 55, 61], and between 30 and 60 min in seven studies [28, 36, 42, 45, 50, 52, 60]. Three studies included other forms of exercise and applied a total training session duration of 45 min [25, 51] or 60 min [46]. In the study by Shimada et al. [39], the duration of the training sessions was individually tailored to each participant. In contrast, 13 other studies did not report the duration of their training sessions at all [35, 37, 38, 41, 43, 44, 48, 53, 56–59, 62]. The duration of perturbation treadmill training ranged from 8.5 to 23 min in seven studies [25, 27, 30, 35, 54, 55, 61], and three additional studies applied 30 min of perturbation training [28, 42, 60]. The remaining 23 studies did not specify the duration of perturbation training.

### Type of perturbation

18 studies focused on walking perturbations [28, 30, 33–35, 37–41, 47, 51–55, 61, 62], and eight studies used standing perturbations [36, 42–44, 49, 50, 58, 60]. The remaining seven studies used a combination of both standing and walking perturbations [25, 27, 29, 31, 45, 46, 59]. Six studies used forward belt acceleration perturbations [29, 42, 43, 50, 58, 60] and eight studies reported the combination of forward and backward belt acceleration perturbations when standing [25, 27, 31, 36, 45, 46, 49, 59]. One study used treadmill belt accelerations [47], five studies used treadmill belt decelerations [33, 39–41, 62], and 15 studies reported a combination of both belt acceleration and deceleration perturbations when walking [25, 27, 31, 34, 35, 37, 38, 45, 46, 51–53, 55, 59, 61]. Seven studies included mediolateral displacement perturbations [27, 31, 35, 45, 46, 52, 55], while another three studies used anteroposterior and mediolateral displacement (but not treadmill belt perturbations; [30, 44, 54]). Two studies incorporated additional tilting perturbations [29, 44], while another study used exclusively three-dimensional tilting movement perturbations [28]. In three studies, mediolateral displacement perturbations were applied with participants standing sideways on the treadmill [25, 36, 58]. Three studies did not report the type of perturbations used [48, 56, 57].

### Predictability of perturbations

A total of 22 studies reported that perturbations were unannounced [22, 30, 31, 34, 36–45, 51–55, 60–62]. One study stated that perturbations were announced only during the first week of the six-week training program and were unannounced from the second week onwards [27]. The remaining twelve studies did not report whether perturbations were announced or unannounced.

16 studies reported that perturbation types were introduced in a randomized order [27, 29–31, 35, 37, 38, 44–46, 50, 52–54, 58, 61]. Two studies varied in their randomization approach: Nørgaard et al. [34] did not randomize perturbation types during the first two sessions but applied randomization in sessions three and four. Hezel et al. [55] randomized perturbation types differently across training blocks: anteroposterior direction in blocks one and two, mediolateral direction in blocks three and four, and all four directions randomized in block five. Two studies introduced two to six random belt decelerations to reduce predictability of belt accelerations [43, 49], while another study did the same without specifying the number of belt decelerations [42]. One study individualized randomization based on the therapist’s judgment [25], and another mentioned varying predictability but did not further define what this meant [57]. Four studies did not report whether perturbation types were randomized.

### Training intensity and progression

20 studies reported individualized adjustments of training intensity, using a variety of criteria. Several studies relied on subjective rating scales: three used a 5-point Likert scale for anxiety and difficulty (with target values between 2–4/5 [27, 55] or > 4/10 [34]), one used a 10-point difficulty scale with a target range of 6–9 [29], and one study combined the Borg scale [6–20] with a 7-point difficulty scale, aiming for values of 12–15 on the Borg scale and ≤ 5 on the difficulty scale [28]. Another study used the Rating of Perceived Exertion (RPE) without specifying a target range [52]. Other adjustment strategies were based on behavioral indicators, such as avoiding the use of handrails [38], the need for a recovery step [44], or demonstrating a successful recovery response in 3 out of 5 perturbations [59]. Four studies described adjustments in general terms: intensity was adapted to provide a constant challenge [30], to tolerate the intensity level [33], or was determined individually by the therapist [35, 42]. Four studies aimed for the maximum intensity at which participants could still recover without falling [31, 43, 54, 58], and one study allowed up to two falls per training block [47]. Wang et al. [40] reported using five different intensity levels, which were increased and then decreased during the training session. Rieger et al. [37] applied two predefined intensity levels. In four studies, initial training intensity was based on baseline assessments: three used the stepping threshold determined during standing perturbations [50, 53, 54], and one applied the so-called Dynamic Stepping Threshold Test [55].

To progressively increase training intensity, most studies adjusted perturbation magnitude (i.e., displacement of the treadmill itself and the treadmill belt), walking speed, or both. Specifically, 21 studies increased perturbation intensity [25, 30, 31, 34–36, 38, 39, 41–44, 46, 47, 49–55]. In three studies, both walking speed and perturbation magnitude were progressively increased [29, 45], and one of those additionally increased the frequency of perturbations [27]. In one further study, walking speed alone was used to progressively increase training intensity [28]. The remaining six studies did not report any information on training intensity adjustments.

### Frequency of perturbations

When the frequency of perturbations was declared, it ranged mostly between every 10 and 60 s and was applied in a randomized order [30, 34, 37, 38, 54, 55, 61], every 2 to 4 strides [52], or as 3 to 5 perturbations per minute [27]. Three additional studies stated that perturbations were applied at randomized frequencies but did not provide further details [53, 58, 60]. The remaining 2 studies did not specify the frequency of perturbations.

### Number of perturbations per training session

Figure 2 provides a visual representation of the number of perturbations observed in the analyzed studies. 12 studies induced between 15 and 40 perturbations [36–38, 42, 43, 48, 49, 53, 54, 58, 61, 62], six studies used between 66 and 160 perturbations [27, 31, 44–46, 50], and six studies used exactly 40 perturbations per training session [33, 34, 40, 47, 55, 62]. Nine studies divided the training sessions into blocks. In five of these studies, each block included between 2 and 12 perturbations [34, 40, 49, 55, 62]. One study structured the sessions into 4-min blocks without specifying the number of perturbations per block [27]. Five other studies reported using between 3 and 11 blocks but did not provide information on either the number of perturbations or the duration per block [28, 40, 47, 53, 59]. 11 studies did not report the number of perturbations per training session.

### Treadmill belt speed

In 20 studies, the treadmill belt speed was predominantly set to the participants’ preferred speed [30, 34, 35, 38, 41, 46, 47, 51, 52, 54]. Two of these studies gave participants a choice of four speed options [33, 62], while one started with a ramp protocol at 0.5 m/s and gradually increased until the participants reached their preferred walking speed [29]. In four other studies, the treadmill speed was determined in other ways, namely using the speed assessed during a pressure-sensitive walkway gait analysis, with a maximum speed deviation of 10% [27], using 70% [28] or 80% [45] of the overground walking speed and by using 50/70% of the maximum treadmill walking speed determined at the beginning of each session [39]. One study fixed the treadmill speed at 1 m/s [37]. Four studies adjusted the walking speed by increasing and decreasing speed to identify the upper and lower limits based on participant feedback and then using the mean between those values [34, 53, 55, 61]. Additionally, five studies reported that researchers frequently varied walking speeds during training [28, 38, 39, 45, 52]. The remaining seven studies did not specify the treadmill belt speed.

### Gait event during perturbation

Perturbations were applied at foot contact [38], mid-stance [27, 31, 55], heel strike [37], immediately after heel strike [53], or randomly throughout the gait cycle [30, 54]. Punt et al. [52] induced perturbations during three gait events (foot contact, mid-stance, and foot off) and Nørgaard et al. [34] even specified the timing of perturbations during different gait events, such as treadmill belt deceleration at heel strike and belt acceleration at mid-swing of the opposite leg. However, the remaining 17 studies did not provide any information about the timing of the perturbation during the gait cycle.

### Perturbed leg

Only five studies mentioned that perturbations were randomized and applied to both legs [37, 38, 53], with two of these stating that perturbations were distributed equally between the legs [34, 55]. Punt et al. [52] used perturbations in both paretic and nonparetic limbs without mentioning whether the distribution was equal between both legs. The remaining 21 studies did not provide any information about the perturbed leg. 

### Theoretical justification

The detailed information and references of the theoretical justifications are presented in Additional file 3. When a theoretical justification was stated, the majority related to general information, predictability of perturbations and training intensity and progression. With regard to general information, eight studies justified their training approach and protocols by referring to previous PBT reviews and studies [27, 31, 40, 42, 50, 55, 58, 62]. Three studies reported including treadmill familiarization prior to PBT training, with their justification based on earlier treadmill research [28, 34, 37]. Nachmani et al. [54] justified their protocol using principles of physical training and exercise prescription, whereas Petrovic et al. [35] stated that no standardized treadmill protocol was feasible due to the multimorbid nature of their population. Punt et al. [52] based their approach on principles of motor learning.

The training period was explicitly justified only in the studies of Punt et al. [52], who based its duration on a previous treadmill study involving participants with stroke, and in the study of Norgaard et al. [34], who based their booster session on a previous PBT study. The duration of the training session was justified by three studies. Gerards et al. [29] based their justification on previous PBT studies, Hezel et al. [55] on the feasibility, while Lurie et al. [25] based their justification of the duration on the feasibility evaluated in their previous pilot study.

The type of perturbation was justified in four studies, all of which referred to previous PBT reviews and studies as their justification [27, 29, 37, 38]. The predictability of perturbations was addressed in 12 studies. Seven justifications were based on the authors’ own assumptions: four aimed to minimize anticipation of perturbations [42–44, 49], two tried to improve ecological validity [30, 33], and one intended to reduce proactive adjustments [27]. Four studies referred to previous PBT research when justifying their approach [45]. Two of those specifically highlighted the use of a random order of perturbations [34, 38], and one cited the application of unannounced perturbations [61]. Nachmani et al. [54] justified their use of a random order of perturbations by referring on principles of motor learning. The training intensity and progression was justified in nine studies. Six of these relied on previous PBT reviews and studies for their justification [28, 33, 34, 37], with two of those additionally modifying the intensity based on the authors’ own experience [29, 49]. Two studies justified their adjustment of the intensity setting using a 5-point Likert scale derived from a previous PBT study [27, 55], and one study based its justification on general principles of physical training [54].

The frequency of perturbations was justified in three studies: two based their decision on the authors’ own experience [27, 52], and one stated that the use of random time intervals was justified from a previous PBT review and study [38].Regarding the number of perturbations, four studies based their justification on previous PBT studies and reviews [33, 34, 38, 45], while one of those additionally used the authors’ own assumptions [62].

The treadmill belt speed was justified in five studies: one referred to a previous PBT study [55], while the other four based their justification on research on treadmill walking [34, 45, 61] and walking speed [38].The gait event was justified only in the study by Brüll et al. [27], who based their choice of gait phase on the recommendations of a previous PBT review, and in the study by Gimmon et al. [30], who applied perturbations in all phases of the gait cycle for ecological validity, based on the authors’ own assumptions. The perturbed leg was justified in only two studies, both referring to a previous PBT review and study [27, 38].

Eleven studies did not provide a justification in any category [36, 39, 41, 47, 51]; however, four of these were only trial registrations [56, 57, 59, 60], one was a preprint [53], and for another, only an abstract was available [48].

## Discussion

The aim of this review was to summarize the available evidence on perturbation treadmill training protocols for falls prevention programs in older adults. Across the 36 included studies, training periods ranged from one to 24 sessions, mostly spread over two to 12 weeks, with one to three sessions per week. Session duration generally varied between 20 and 60 min. Most studies applied unannounced perturbations and used perturbation types in randomized order, primarily consisting of belt acceleration and deceleration during both standing and walking. The number of perturbations per session ranged widely from 15 up to 160 perturbations. Training intensity was often individually adjusted, and the treadmill speed for walking perturbations was commonly adjusted to the participant's preferred walking speed. Parameters like the frequency of perturbations, gait events during perturbations, or the perturbed leg, were rarely reported and little information was provided on the theoretical justification of the training protocol features.

The substantial heterogeneity of PBT protocols, in conjunction with the predominance of studies published in 2019 or later, demonstrates that this field of research is still in a relatively early stage of development. It is evident that a consensus on the optimal training design has yet to be achieved, also limiting the ability to conduct robust meta-analyses. The ideal training dosage is the subject of a growing number of studies. The range of training volumes examined extends from several months of PBT to interventions in which participants only perform a single PBT session [33, 96]. However, the question remains as to whether a greater number of training sessions [97] and perturbations [32, 98] or a booster session after several months [96] may be necessary for this approach. Karamanidis et al. [99] for example introduced a critical threshold for perturbation dose, defined as the number of trials with a minimum of eight perturbations and a maximum of 24 perturbations. This threshold has to be accomplished for long-term retention of training effects, is individually different and affected by sensory and neuromotor pathologies.

In order to achieve the best possible training effects, it is essential to critically discuss not only the optimal dosage of PBT, but also its ecological validity. PBT, considered as a task-specific approach to falls prevention, aims at not only improving predictive motor adaptations (i.e., proactive adjustment of balance control during movement), but also, and most importantly, reactive adaptations. By explicitly targeting destabilization and the need for a sudden reaction to regain balance, the motor response to external perturbations is trained. This encompasses the initiation of the balance recovery responses and the optimization of motor programs and intermuscular coordination [17]. Since falls in everyday life do happen unannounced, the predictability of perturbations is an aspect that should be highlighted regarding the ecological validity of PBT. Most of the reviewed studies reported on this parameter and induced perturbations unannounced and/or in randomized order regarding the direction of perturbations. Anticipatory adjustments can be reduced this way to center on reactive recovery responses [21]. In this regard, treadmills assume a unique role when contrasted with overground setups, for instance, due to their capacity to introduce perturbations in an unpredictable manner. Nevertheless, the generalizability of treadmill perturbations to real-life fall situations remains a point of discussion requiring further research. The biomechanical characteristics of a perturbation induced by a surface translation on the treadmill do not perfectly simulate an obstacle-induced overground trip [98]. Despite the inherent limitations, multiple RCTs report encouraging results for treadmill-based PBT, including reductions in falls and fall-related risk measures [18]. Together with the practical advantages offered by treadmills for conducting reactive balance training, especially in clinical settings, this approach still plays an important role in PBT research.

In terms of ecological validity, one further aspect to consider is the application of perturbations during standing versus walking. Most of the included studies used the latter approach, but there were also several interventions that consisted solely of static trials or a combination of both. The reviews of Gerards et al. [22] and McCrum et al. [17] discuss this aspect, providing arguments for both methods depending on the frailty or mobility of the target population, but beyond practical considerations, there is little evidence on how this affects overall effectiveness of the training. Furthermore, falls in everyday life are not limited to a single plane but rather can involve rotational movements [100] which is hard to reconstruct on a treadmill. The perturbation types incorporated in the included studies varied greatly, with less than half of them challenging reactive balance in the anteroposterior as well as mediolateral direction. With regard to falls prevention training, it is still unclear, whether transfer from one perturbation type to another is possible [37]. However, not all treadmills can simulate lateral perturbations during walking. Usually, the entire device needs to be displaced sideways, which requires special technical features. This once again highlights the fact that in clinical research and practice, there is often a discrepancy between theoretically ideal conditions and the practical limitations imposed by the available resources. When decisions regarding the design of an intervention are made for pragmatic rather than theoretical reasons, this should be explicitly stated in the study.

The majority of the included studies implemented perturbation-based training at participants’ self-selected walking speed on a treadmill. While this approach may enhance comfort and feasibility, walking behavior differs between treadmill and overground conditions and is further influenced by environmental context [101]. Consequently, findings derived under one condition should not be readily transferred to another without careful consideration. Moreover, gait speed is a key determinant of dynamic stability and directly influences the temporal constraints within which reactive balance responses must be generated. Variations in walking speed therefore have important implications for both the stability demands imposed by perturbations and the nature of the resulting balance recovery strategies.

There were significant gaps in reporting on gait phase and the perturbed leg, which were addressed in only 41.7% and 52.8% of the articles analyzed, respectively. However, it is imperative to provide a precise definition of these aspects when designing perturbation-based training protocols. For example, balance recovery strategies differ substantially depending on the gait phase in which an external perturbation occurs, such as during early versus late swing [102]. Accordingly, the timing of perturbations should be carefully considered in the design of PBT protocols, as it represents a key determinant of the elicited balance responses and should reflect, as far as feasible, the diversity and complexity of balance challenges encountered in real-life walking situations.

Another parameter that exhibits considerable heterogeneity and limited justification in the articles analyzed is the training intensity. In most cases, the magnitude of the perturbations was used to regulate the intensity, although in some cases, the walking speed was also considered. Other factors that could affect training intensity include the number of perturbations in a session, fatigue, the use of physical or cognitive dual task exercises, the knowledge the participants have about the training protocol and the variation of perturbation type as well as the level of predictability within the protocol. This great variability demonstrates the complexity of the subject and the difficulties inherent in formulating a uniform definition and measurement of perturbation intensity. Similar challenges were reported by Farlie et al. [103]. These methods differ in their level of objectivity and reproducibility, emphasizing the need for further standardization. A clear and comprehensive definition of training intensity in PBT would facilitate future research by enabling a more systematic investigation of dose–response relationships, provided that the factors influencing intensity are clearly identified and their interaction and controllability are well understood. Moreover, from a clinical perspective, such a definition is essential for selecting an appropriate training load to ensure that an effective and sufficiently challenging training stimulus is applied.

Furthermore, it is not only crucial to provide comprehensive reporting of *what* was done in an intervention, but also to state *why* training parameters were designed the way they were. Knowing the underlying justification, whether it is based on theoretical evidence or more practical aspects, can, for one thing, support clinicians in the implementation process of PBT protocols. On the other hand, it helps other researchers to understand for which parameters evidence already exists and on which aspects research gaps are still remaining. This review revealed that in the included studies the justification for PBT protocol features was infrequently mentioned or only stated for some training parameters. This may partly be due to inadequate reporting, as mentioned above. However, this may also indicate that there is still insufficient underlying evidence for some parameters. General information on training parameters, the predictability of perturbations as well as training intensity and progression were the PBT protocol features whose justification was reported most frequently and which were commonly based on general motor learning principles or previous PBT studies. However, such justifications often appeared to rely primarily on precedent rather than on evidence supporting the effectiveness or optimality of specific design choices. This suggests that current protocol features may, at least in part, be shaped by pragmatic considerations or by the absence of robust evidence regarding the optimal dose–response relationship, rather than by systematically derived empirical findings. As the body of literature on experimental and interventional studies on PBT continues to grow, the number of reviews in this field is also expanding [17, 21, 22, 104, 105]. Articles like these, which provide concentrated information on the subject, can serve as a valuable source in the process of designing an intervention based on the current evidence and can help to justify the chosen method. Nonetheless, there is still great potential for future research to take a closer look at various PBT features such as dose–response relationships, the effect of instruction (e.g., how much should participants know in advance about the protocol), the perturbation schedule (e.g. block, randomized, etc.) or retention and generalization effects of PBT.

### Recommendations for reporting PBT protocols

This review highlights the insufficient reporting of training parameters and their justification. The lack of transparency in setting the training parameters makes it difficult to discuss results and compare them with other studies, as well as posing a challenge for implementation in evidence-based practice. Therefore, we propose recommendations for protocol reporting for PBT (ProRePBT), as shown in Table 3. These can be used in addition to established general reporting tools for exercise interventions like the TIDieR [23] or the CERT [24]. The first seven items of the proposed PBT-specific reporting checklist can be applied to both standing and walking perturbation paradigms. To address the specific requirements of walking perturbations, there are three additional items included. As previously stated, it would further be of great value for other researchers and health care providers to get insight into the justification for the protocol features, why this should be reported as well. The proposed items are based on the results of this review on treadmill PBT, and therefore include some treadmill-specific items. Nevertheless, the item set can also be adapted to other perturbation methods.

**Table 3:** Recommendations for protocol reporting for PBT (ProRePBT)

Item number	Item	Description of item	Location where item is reported
1	Perturbation treadmill	Specify the treadmill used for PBT, including key technical specifications and capabilities to induce perturbations (e.g., belt acceleration/deceleration, lateral shift, tilting platform). Indicate the presence of additional measurement systems, such as integrated force plates or motion sensors, and describe their role in detecting gait events (e.g., perturbation onset timing)	
2	Duration of total training session; duration of perturbation training	Specify the total duration of each training session, including additional exercise components and treadmill familiarization when applicable. If possible, also report the precise time dedicated specifically to perturbation exposure	
3	Type of perturbation	Describe the perturbation type(s) applied (e.g. belt acceleration/deceleration, lateral displacement, platform pitching or tilting) and specify whether perturbations were delivered during standing and/or walking. If applicable, state the direction in which participants are standing on the treadmill (e.g. facing an integrated screen or with their back to it, positioned sideways on the treadmill belt). If possible, provide sufficient detail on the characteristics of each perturbation type to enable replication (e.g., direction, displacement, velocity change, and, where applicable, the perturbation level specific to the treadmill used)	
4	Number of perturbations per training session	Provide the total number of perturbations applied in each session, including, if applicable, the specific number for each perturbation type. Additionally, report whether perturbations were divided into blocks with rest breaks in between, and specify the number of perturbations per block	
5	Predictability of perturbations	Indicate whether perturbations were delivered in a predictable or unpredictable manner. If unpredictable, describe how unpredictability was achieved (e.g., random timing or random order of perturbation types), and specify whether participants received any cues or announcements regarding the timing or type of upcoming perturbations	
6	Frequency of perturbations	Specify the time interval between perturbations (e.g., delivering perturbations every X seconds, steps, or strides)	
7	Training intensity and progression	Describe the intensity of perturbations and how it was adjusted (e.g., by changing perturbation magnitude, belt speed, frequency of perturbations, or incorporating additional dual tasks), either according to predefined increments and protocols or individually based on participant performance. If applicable, specify the method of intensity progression, such as using objective measures (e.g., stepping thresholds, center of mass metrics) or subjective assessments (e.g., scales, questionnaires rating perceived difficulty)	
Additional items for walking perturbations			
8	Walking speed	Specify the walking speed during perturbation training, indicating whether the speed was fixed or self-selected. If applicable, report how walking speed was determined (and for treadmill PBT, whether it was based on treadmill or overground walking). Additionally, indicate if walking speed was adapted or changed during the training period	
9	Gait event during perturbation	Describe at which gait phase the perturbation was delivered (e.g., heel strike, mid-stance, foot off) and how the timing was determined, or if another method was used	
10	Perturbed leg	Indicate whether perturbations targeted the left leg, right leg, or both. If both legs were targeted, specify whether perturbations were applied equally to each leg and whether leg selection was randomized or alternate	

While these recommendations were developed based on PBT on treadmills, they are also applicable to other types of PBT, such as overground or cable-pull perturbations

All items included in the ProRePBT checklist represent key control elements of PBT and are therefore essential for the appropriate prescription, interpretation, and replication of PBT interventions. As demonstrated by the findings of this review, several fundamental parameters were reported inconsistently across the reviewed studies. For example, Item 6 (frequency of perturbations) was explicitly reported in only 36.1% of the included studies, while parameters such as Item 4 (gait event during perturbation) and Item 5 (perturbed leg) were likewise infrequently specified. In addition, the substantial heterogeneity and often limited justification regarding Item 7 (training intensity and progression) further emphasize the need for systematic and standardized reporting of these elements to enable meaningful comparison across studies and to support clinical implementation.

Despite the heterogeneity of training protocols revealed in this review, there is evidence indicating that PBT might be an effective approach for improving reactive balance and reducing falls [18, 21, 106]. Further investigation of the dose–response relationships involved is necessary to create PBT protocols that are both effective and resource efficient. It is crucial to further evaluate which parameters have the most influence on effectiveness and develop systematic approaches to adjust the training to the individual needs of the target population. Future research would benefit from advanced study designs that allow systematic evaluation of multiple PBT components and their interactions. Adaptive approaches, such as Sequential Multiple Assignment Randomized Trials (SMART), could help iteratively adjust training parameters based on individual responses, providing insight into how protocols can be optimized and tailored to participant needs. Such strategies may ultimately support the development of more effective, efficient, and personalized PBT interventions. When designing training protocols for older adults with neurological disorders, it is important to consider that the underlying neurophysiological mechanisms may differ from those of healthy older populations [99].

### Limitations

The majority of the included studies focus on healthy older adults or those at risk of falling. Only ten studies with neurological patients met the eligibility criteria for this review. In many cases, studies for this target group were experimental or the study population had a mean age of less than 60 years. It is important to note that some neurological conditions, such as Parkinson's disease, stroke and especially multiple sclerosis, may start at an earlier age than the already lowered expected cutoff age that was used as an inclusion criterion. Another limitation of this review is that we solely focused on perturbations induced by the treadmill itself. This was done to narrow down the research question and to better summarize training protocols. However, there are many other ways to initiate perturbations during treadmill training, such as manual perturbations, cable pulls, obstacles, and visual or acoustic cues. These methods could be of importance for further research. Moreover, this review does not allow conclusions to be drawn about the effectiveness of training protocol features, as no assessment of study quality or further analyses were conducted. The aim was rather to provide an overview of current approaches and identify gaps in reporting of training parameters. Finally, it should be considered that some of the included studies were only available as trial registry entries. The amount of detail provided on methodology varies greatly between study registrations, and in some cases only limited information could be extracted for this review.

## Conclusions

This review reveals a high heterogeneity in training protocols used in the emerging field of PBT. Additionally, a comprehensive and detailed documentation of all relevant parameters and the theoretical justification of the training protocol is often lacking. Therefore, we propose recommendations for protocol reporting for PBT (ProRePBT) for more comprehensive reporting of training parameters to increase the comparability of study results and facilitate the implementation of PBT into clinical practice. Future studies should further investigate PBT features like perturbation types and their generalizability, dose–response relationships or the perturbation schedule to support systematic, targeted, and efficient training.

## Supplementary Information

Supplementary Material 1.

Supplementary Material 2.

Supplementary Material 3.

Supplementary Material 4.

## Data Availability

A detailed list of excluded studies with reasons for exclusion is available from the corresponding author upon reasonable request.

## Abbreviations

ProRePBT: Protocol reporting for perturbation-based balance training
PBT: Perturbation-based balance training
TIDieR: Template for Intervention Description and Replication
CERT: Consensus on Exercise Reporting Template
PICO: Population, intervention, comparison, outcome
RCT: Randomized Controlled Trial
IQR: Interquartile Range
BaMPer System: Balance Measure and Perturbation System
GRAIL: Gait Real-time Analysis Interactive Lab
CAREN: Computer Assisted Rehabilitation Environment
RPE: Rating of Perceived Exertion
