# Supplementary material for: Perturbation-based balance training on treadmills for falls prevention in older adults: a review of training protocols and reporting recommendations (ProRePBT)
Source: BMC Geriatr. 2026 Feb 14;26:300. doi: 10.1186/s12877-026-07124-3 (PMC12958537; doi:10.1186/s12877-026-07124-3)
Supplement: Supplementary file 1 — Supplementary Material 1. [file 12877_2026_7124_MOESM1_ESM.docx]

**Additional file 1.**

Detailed search strategies:

CENTRAL

09/05/2025

161 Results

No filters

#1 MeSH descriptor: [Stroke] explode all trees

#2 (stroke):ti,ab,kw OR (apoplexy):ti,ab,kw OR (“cerebrovascular accident”):ti,ab,kw OR (“brain infarction”):ti,ab,kw OR (“brain ischemia”):ti,ab,kw OR (“brain attack”):ti,ab,kw OR (“Cerebral infarction”):ti,ab,kw OR (“Intracranial hemorrhage”):ti,ab,kw (Word variations have been searched)

#3 (Parkinson*):ti,ab,kw OR (PD):ti,ab,kw (Word variations have been searched)

#4 (“Multiple sclerosis”):ti,ab,kw OR (MS):ti,ab,kw (Word variations have been searched)

#5 (“Old adults”):ti,ab,kw OR (older):ti,ab,kw OR (Elder*):ti,ab,kw OR (Senior*):ti,ab,kw OR (Aged):ti,ab,kw OR (ageing):ti,ab,kw OR (aging):ti,ab,kw (Word variations have been searched)

#6 #1 OR #2 OR #3 OR #4 OR #5

#7 ((perturbation):ti,ab,kw OR (slip):ti,ab,kw OR (trip):ti,ab,kw OR (PBBT):ti,ab,kw OR (PBT):ti,ab,kw OR (“reactive balance”):ti,ab,kw) AND (treadmill):ti,ab,kw (Word variations have been searched)

#8 (Balance):ti,ab,kw OR (“Reactive recovery response”):ti,ab,kw OR (“Postural control”):ti,ab,kw OR (Stability):ti,ab,kw OR (gait):ti,ab,kw OR (mobility):ti,ab,kw OR (“physical capacity”):ti,ab,kw OR (“functional capacity”):ti,ab,kw OR (“physical functioning”):ti,ab,kw OR (Fall*):ti,ab,kw (Word variations have been searched)

#9 #6 AND #7 AND #8

CINAHL

09/05/2025

96 Results

No filters

XB (stroke OR apoplexy OR “cerebrovascular accident” OR “brain infarction” OR “brain ischemia” OR “brain attack” OR “Cerebral infarction” OR “Intracranial hemorrhage” OR Parkinson* OR PD OR “Multiple sclerosis” OR MS OR “Old adults” OR older OR Elder* OR Senior* OR Aged OR ageing OR aging) AND XB ((Perturbation OR slip OR trip OR PBBT OR PBT OR “reactive balance”) AND treadmill) AND XB (Balance OR “Reactive recovery response” OR “Postural control” OR Stability OR gait OR mobility OR “physical capacity” OR “functional capacity” OR “physical functioning” OR Fall*)

Embase

09/05/2025

320 Results

No filters

#1 stroke:ti,ab,kw OR apoplexy:ti,ab,kw OR 'cerebrovascular accident':ti,ab,kw OR 'brain infarction':ti,ab,kw OR 'brain ischemia':ti,ab,kw OR 'brain attack':ti,ab,kw OR 'cerebral infarction':ti,ab,kw OR 'intracranial hemorrhage':ti,ab,kw

#2 parkinson*:ti,ab,kw OR pd:ti,ab,kw

#3 'multiple sclerosis':ti,ab,kw OR ms:ti,ab,kw

#4 'old adults':ti,ab,kw OR older:ti,ab,kw OR elder*:ti,ab,kw OR senior*:ti,ab,kw OR aged:ti,ab,kw OR ageing:ti,ab,kw OR aging:ti,ab,kw

#5 #1 OR #2 OR #3 OR #4

#6 (perturbation:ti,ab,kw OR slip:ti,ab,kw OR trip:ti,ab,kw OR pbbt:ti,ab,kw OR pbt:ti,ab,kw OR 'reactive balance':ti,ab,kw) AND treadmill:ti,ab,kw

#7 balance:ti,ab,kw OR 'reactive recovery response':ti,ab,kw OR 'postural control':ti,ab,kw OR stability:ti,ab,kw OR gait:ti,ab,kw OR mobility:ti,ab,kw OR 'physical capacity':ti,ab,kw OR 'functional capacity':ti,ab,kw OR 'physical functioning':ti,ab,kw OR fall*:ti,ab,kw

#8 #5 AND #6 AND #7

PubMed

10/05/2025

290 Results

No filters

#1 "Stroke"[MeSH Terms] OR "Stroke"[Title/Abstract] OR "apoplexy"[Title/Abstract] OR "cerebrovascular accident"[Title/Abstract] OR "brain infarction"[Title/Abstract] OR "brain ischemia"[Title/Abstract] OR "brain attack"[Title/Abstract] OR "cerebral infarction"[Title/Abstract] OR "intracranial hemorrhage"[Title/Abstract]

#2 "Parkinson Disease"[MeSH Terms] OR "parkinson*"[Title/Abstract] OR "PD"[Title/Abstract]

#3 "Multiple Sclerosis"[MeSH Terms] OR "Multiple Sclerosis"[Title/Abstract] OR "MS"[Title/Abstract]

#4 "Aged"[MeSH Terms] OR "old adults"[Title/Abstract] OR "older"[Title/Abstract] OR "elder*"[Title/Abstract] OR "senior*"[Title/Abstract] OR "Aged"[Title/Abstract] OR "ageing"[Title/Abstract] OR "aging"[Title/Abstract]

#5 #1 OR #2 OR #3 OR #4

#6 "perturbation"[Title/Abstract] OR "slip"[Title/Abstract] OR "trip"[Title/Abstract] OR "PBBT"[Title/Abstract] OR "PBT"[Title/Abstract] OR "reactive balance"[Title/Abstract]

#7 "treadmill"[Title/Abstract]

#8 "Postural Balance"[MeSH Terms] OR "balance"[Title/Abstract] OR "reactive recovery response"[Title/Abstract] OR "postural control"[Title/Abstract] OR "stability"[Title/Abstract] OR "gait"[Title/Abstract] OR "mobility"[Title/Abstract] OR "physical capacity"[Title/Abstract] OR "functional capacity"[Title/Abstract] OR "physical functioning"[Title/Abstract]

#9 "Accidental Falls"[MeSH Terms] OR "fall*"[Title/Abstract]

#10 #8 OR #9

#11 #5 AND #6 AND #7 AND #10

Web of Science

10/05/2025

353 Results

No filters

((AB=(stroke OR apoplexy OR "cerebrovascular accident" OR "brain infarction" OR "brain ischemia" OR "brain attack" OR "cerebral infarction" OR "intracranial hemorrhage" OR parkinson* OR PD OR "multiple sclerosis" OR MS OR "old adults" OR older OR elder* OR senior* OR aged OR ageing OR aging)) AND AB=((perturbation OR slip OR trip OR PBBT OR PBT OR "reactive balance") AND treadmill)) AND AB=(balance OR "reactive recovery response" OR "postural control" OR stability OR gait OR mobility OR "physical capacity" OR "functional capacity" OR "physical functioning" OR fall*)

ClinicalTrials.gov

10/05/2025

33 Results

No filters

Condition/disease:
stroke OR apoplexy OR "cerebrovascular accident" OR "brain infarction" OR "brain ischemia" OR "brain attack" OR "cerebral infarction" OR "intracranial hemorrhage" OR parkinson* OR PD OR "multiple sclerosis" OR MS OR "old adults" OR older OR elder* OR senior* OR aged OR ageing OR aging

Other terms:
balance OR "reactive recovery response" OR "postural control" OR stability OR gait OR mobility OR "physical capacity" OR "functional capacity" OR "physical functioning" OR fall*

Intervention/treatment:
perturbation OR slip OR trip OR PBBT OR PBT OR "reactive balance") AND treadmill
