# Supplementary material for: Perturbation-based balance training on treadmills for falls prevention in older adults: a review of training protocols and reporting recommendations (ProRePBT)
Source: BMC Geriatr. 2026 Feb 14;26:300. doi: 10.1186/s12877-026-07124-3 (PMC12958537; doi:10.1186/s12877-026-07124-3)
Supplement: Supplementary file 2 — Supplementary Material 2. [file 12877_2026_7124_MOESM2_ESM.docx]

**Additional file 2.**

Table A1. *Overview of the training parameters: perturbation treadmill, training period and number of training sessions, training session duration, type and predictability of perturbations.*

| **Study** | **Perturbation treadmill** | **Number of training sessions and training period** | **Duration of total training session; duration of perturbation training** | **Type of perturbation** | **Predictability of perturbations (announcement of perturbations; randomization of perturbation types)** |
| --- | --- | --- | --- | --- | --- |
| Allin et al., 2020 (1) | Modified treadmill (not further specified) | 4 (2x/week for 2 weeks) | 30-60 minutes; 30 minutes | Forward belt acceleration when standing | Unannounced perturbations; including belt decelerations pseudo-randomly to reduce predictability (number not specified) |
| Aviles et al., 2019 (2) | Freemotion 800 (Freemotion Fitness, Logan, UT, United States) | 12 (3x/week for 4 weeks) | 30 minutes; NR | Forward and backward belt acceleration when standing | NR; including two belt decelerations to reduce predictability |
| Bhatt et al., 2018 (3) | NR | INT1: 3 (1x/day) INT2: 4 (1x/week for 4 weeks) | NR; NR | Forward and backward belt acceleration when standing, belt acceleration and deceleration when walking | NR; NR |
| Brüll et al., 2023 (4) | BalanceTutor™ (MediTouch LTD, Netanya, Israel) | 18 (3x/week for 6 weeks) | 24 minutes; 4 blocks of 4 minutes each | Forward and backward belt acceleration when standing, belt acceleration and deceleration when walking, lateral displacement when standing and walking | Announced perturbations in week one, unannounced in following weeks; perturbation types in randomized order |
| Cheng et al., 2020 (5) | QQ-Mill (Motekforce Link, Amsterdam, The Netherlands) | 8 (2x/week for 4 weeks) | 60 minutes; NR | Forward and backward belt acceleration when standing, belt acceleration and deceleration when walking, lateral displacement when standing and walking | Unannounced perturbations; perturbation types in randomized order |

Table A1. *Continued.*

| **Study** | **Perturbation treadmill** | **Number of training sessions and training period** | **Duration of total training session; duration of perturbation training** | **Type of perturbation** | **Predictability of perturbations (announcement of perturbations; randomization of perturbation types)** |
| --- | --- | --- | --- | --- | --- |
| Chien et al. 2018 (6) | Custom-made split belt treadmill (not further specified) | 16 (2x/week for 8 weeks) | 60 minutes including warm-up and stretching exercises; NR | Forward and backward belt acceleration when standing, belt acceleration and deceleration when walking, lateral displacement when standing and walking | NR; perturbation types in randomized order |
| Dusane et al., 2021 (7) | ActiveStep (Simbex, Lebanon, NH, United States) | 4 (1x/week for 4 weeks) | NR; NR | Belt acceleration when walking | NR; NA |
| Faria et al., 2023 (8) | Commercial treadmill LX160 (Movement, Pompéia - SP, Brazil), customized with a motor (Weg 22 Plus) | INT1: 4 (2x/week for 2 weeks) & INT2: 4 (2x/day with a 24-hour break in between the first two and the last two sessions) | INT1 & INT2: 20-25min; 18-23min | Belt acceleration and deceleration when walking | Unannounced perturbations; perturbation types in randomized order |
| Gassner et al. 2019 (9) | Medical treadmill (h/p/cosmos Sports & Medical GmbH, Nussdorf, Germany) mounted on a tiltable platform construction (Zebris Medical GmbH, Isny, Germany) | 8 (2x/week for 8-9 weeks) | 40 minutes; 30 minutes | Three-dimensional tilting movements when walking | NR; NA |
| Gerards et al., 2023 (10) | Computer Assisted Rehabilitation Environment (CAREN; Motek Medical B.V., Houten, The Netherlands) | 3 (1x/week for 3 weeks) | 30 minutes; NR | Forward belt acceleration when standing, belt acceleration and deceleration and lateral displacement when walking, shifting and tilting when standing and walking | Unannounced perturbations; perturbation types in randomized order |

Table A1. *Continued.*

| **Study** | **Perturbation treadmill** | **Number of training sessions and training period** | **Duration of total training session; duration of perturbation training** | **Type of perturbation** | **Predictability of perturbations (announcement of perturbations; randomization of perturbation types)** |
| --- | --- | --- | --- | --- | --- |
| Gimmon et al., 2018 (11) | Balance Measure and Perturbation System (BaMPer) (12) | 24 (2x/week for 12 weeks) | 20 minutes; 14 minutes | Anteroposterior and lateral displacement when walking | Unannounced perturbations; perturbation types in randomized order |
| Grabiner et al., 2012 (13) | ActiveStep (Simbex, Lebanon, NH, United States) | 4-10 (in 3.5 – 4 weeks) | NR; NR | Forward belt acceleration when standing | Unannounced perturbations; including belt decelerations randomly to reduce predictability (3 times for sessions with 15 trials, 6 times for sessions with 30 trials) |
| Handelzalts et al., 2019 (14) | BalanceTutor™ (MediTouch LTD, Netanya, Israel) | 12 (1x/day for 2.5 weeks) | 30 minutes; NR | Forward and backward belt acceleration when standing, lateral displacement when standing and walking | Unannounced perturbations; perturbation types in randomized order |
| Hezel et al., 2023 (15) | BalanceTutor™ (MediTouch LTD, Netanya, Israel) | INT1: 6 (1x/week for 6 weeks), INT2: 2 (one session in week 1 and one in week 6) | 30 minutes; 8.5-14.5 minutes | Belt acceleration and deceleration, lateral displacement when walking | Unannounced perturbations; block 1+2: AP direction in randomized order; block 3+4: ML direction in randomized order; block 5: all four directions in randomized order |
| Lanza et al., 2024 (16) | ActiveStep (Simbex, Lebanon, NH, United States) | 18 (3x/week for 6 weeks) | 45 minutes; NR | Forward belt acceleration when standing | NR; perturbation types in randomized order |
| Lee et al., 2018 (17) | ActiveStep (Simbex, Lebanon, NH, United States) | Single session | NR; NR | Belt deceleration when walking | Unannounced perturbations; NA |
| Liu et al., 2021 (18) | ActiveStep (Simbex, Lebanon, NH, United States) | Single session | 30 minutes; NR | Belt deceleration when walking | NR; NA |

Table A1. *Continued.*

| **Study** | **Perturbation treadmill** | **Number of training sessions and training period** | **Duration of total training session; duration of perturbation training** | **Type of perturbation** | **Predictability of perturbations (announcement of perturbations; randomization of perturbation types)** |
| --- | --- | --- | --- | --- | --- |
| Lurie et al., 2013 (19) | ActiveStep (Simbex, Lebanon, NH, United States) | 1-19 sessions (M = 5.8) | M = 44.3 minutes including exercises for strengthening and flexibility, static and dynamic balance, and mobility training; NR | Belt acceleration and deceleration when walking | Unannounced perturbations; NR |
| Lurie et al., 2020 (20) | ActiveStep (Simbex, Lebanon, NH, United States) | 8-18 (2-3x/week for 4-6 weeks) | 45 minutes including exercises for strengthening and flexibility, static and dynamic balance, and mobility training; 15 minutes | Forward and backward belt acceleration when standing, belt acceleration and deceleration when walking, occasional lateral displacement (induced when standing sideways) | NR; individualized randomization based on therapist’s opinion |
| Montana State University, 2021 (21) | NR | 24 (2x/week for 12 weeks) | NR; NR | Forward belt acceleration when standing, lateral displacement (induced when standing sideways) | NR; perturbation types in randomized order |
| Nachmani et al., 2021 (22) | Balance Measure and Perturbation System (BaMPer) (12) | 8 (2x/week for 4 weeks) | 20-30 minutes; 14-20 minutes | Anteroposterior and lateral displacement when walking | Unannounced perturbations; perturbation types in randomized order |
| Nørgaard et al., 2023 (23) | Split 70/157/ASK (Woodway, Weil am Rhein, Germany) | 4 (two initial on the same day, one in week 3, one in week 26) | 20 minutes; NR | Belt acceleration and deceleration when walking | Unannounced perturbations; session 1+2: perturbation types not in randomized order, session 3+4: perturbation types in randomized order |
| Petrovic et al., 2024 (24) | BalanceTutor™ (MediTouch LTD, Netanya, Israel) | ≥4 sessions within 2 weeks of hospital stay | NR; 15 minutes (partially divided into three 5-minute blocks) | Belt acceleration and deceleration, lateral displacement when walking | NR; perturbation types in randomized order |

Table A1. *Continued.*

| **Study** | **Perturbation treadmill** | **Number of training sessions and training period** | **Duration of total training session; duration of perturbation training** | **Type of perturbations** | **Predictability of perturbations (announcement of perturbations; randomization of perturbation types)** |
| --- | --- | --- | --- | --- | --- |
| Protas et al., 2005 (25) | NR | 24 (3x/week for 8 weeks) | 60 minutes; NR | Forward and backward belt acceleration when standing, lateral displacement (induced when standing sideways) | Unannounced perturbations; perturbation types not randomized (belt acceleration always followed by belt deceleration) |
| Punt et al., 2019 (26) | Gait Real-time Analysis Interactive Lab (GRAIL; Motek Medical B.V., Houten, The Netherlands) | 10 sessions in 6 weeks | 30-60 minutes; NR | Belt acceleration and deceleration, lateral displacement when walking | Unannounced perturbations; perturbation types in randomized order |
| Rieger et al., 2020 (27) | Gait Real-time Analysis Interactive Lab (GRAIL; Motek Medical B.V., Houten, The Netherlands) | 2 (one week between the sessions) | NR; NR | Belt acceleration and deceleration when walking | Unannounced perturbations; perturbation types in randomized order |
| Rieger et al., 2024 (28) | C-Mill VR+ (Motek Medical B.V., Houten, The Netherlands) | 8 (2x/week for 4 weeks) | NR; NR | Belt acceleration and deceleration when walking | Unannounced perturbations; perturbation types in randomized order |
| Shimada et al., 2004 (29) | Bilateral separated treadmill system for gait perturbation (KOROBU-KUN; not further specified) | Total of 600 minutes of training (1-3x/week over 6 months) | Individually tailored (according to rises in blood pressure, complaints of fatigue); NR | Belt deceleration when walking | Unannounced perturbations; NA |
| US Department of Veterans Affairs, 2008 (30) | Custom-built treadmill (not further specified) | 4 (1x/week for 4 weeks) | Progressively increasing perturbation intensity | Belt deceleration when walking | Unannounced perturbations; NA |
| Van Wouwe et al., 2021 (31) | Computer Assisted Rehabilitation Environment (CAREN; Motek Medical B.V., Houten, The Netherlands) | 7 (in 3 weeks) | NR; NR | Anteroposterior and lateral displacement and tilting when standing | Unannounced perturbations; perturbation types in randomized order |

Tabelle A1. *Continued.*

| **Study** | **Perturbation treadmill** | **Number of training sessions and training period** | **Duration of total training session; duration of perturbation training** | **Type of perturbations** | **Predictability of perturbations (announcement of perturbations; randomization of perturbation types)** |
| --- | --- | --- | --- | --- | --- |
| Virginia Polytechnic Institute and State University, 2022 (32) | NR | 6 (2x/week for 3 weeks) | 30-60 minutes; 30 minutes | Forward belt acceleration when standing | Unannounced perturbations; NA |
| Wang et al., 2022 (33) | ActiveStep (Simbex, Lebanon, NH, United States) | Single session | 30 minutes; NR | Belt deceleration when walking | Unannounced perturbations; NA |
| Whitten et al., 2023 (34) | NR | 6 (1x/week for 6 weeks) | NR; NR | NR | NR; NR |
| Yang et al., 2021 (35) | NR | Single session | NR; NR | NR | NR; NR |
| Zhu et al., 2025 (36) | Commercial treadmill Quasar (h/p/cosmos Sports & Medical GmbH, Nussdorf-Traunstein, Germany) | Single session | NR; NR | Belt acceleration and deceleration when walking | Unannounced perturbations; perturbation types in randomized order |
| Zieschang et al., 2024 (37) | NR | 9 sessions | NR; NR | NR | NR; varying predictability (not further defined) |

M: Mean; NA: not applicable (the training parameter is not applicable to the chosen perturbation modality); NR: not reported (no information about the training parameter is given in the study, although the parameter applies to the perturbation modality); INT: Intervention.

*Note.* Only the main publication is listed. For additional related publications, see Table 2.

Table A2. *Overview of the training parameters: training intensity and progression, perturbation frequency, number of perturbations, treadmill belt speed, gait event during perturbation and perturbed leg.*

| **Study** | **Training intensity and progression** | **Frequency of perturbations** | **Number of perturbations per training session** | **Treadmill belt speed (walking perturbations)** | **Gait event during perturbation (walking perturbations)** | **Perturbed leg (walking perturbations)** |
| --- | --- | --- | --- | --- | --- | --- |
| Allin et al., 2020 (1) | Adjusted individually: evaluated qualitatively and visually by trainer, progressively increasing perturbation intensity | NR | 20-30 perturbations | NA | NA | Initial stepping limb was specified and varied by trainer (standing perturbation) |
| Aviles et al., 2019 (2) | Varying in walking speed after perturbation which is induced in approximately 40 milliseconds: acceleration 0.5-2.4 mph and deceleration 0.5 mph, progressively increasing perturbation speed | NR | Up to 40 perturbations, rest break every 10 perturbations | NA | NA | NA |
| Bhatt et al., 2018 (3) | Adjusted individually: intensity will be increased until participant shows a recovery response in 3/5 perturbations | NR | 5 perturbations per block, number of blocks NR | NR | NR | NR |
| Brüll et al., 2023 (4) | Adjusted individually: 5-point scale of subjectively perceived difficulty (3/5) and anxiety (3/5), progressively increasing perturbation intensity, frequency and walking speed | 3-5 perturbations/ minute | 75 ± 14 perturbations, divided into 4 blocks of 4 minutes each | Assessed in pressure-sensitive walkway gait analysis (GAITRite) with mean of three trials and a max. speed deviation of 10% | Mid-stance | NR |
| Cheng et al., 2020 (5) | Progressively increasing perturbation intensity and walking speed | NR | 80 perturbations | 80% of overground walking speed (10m gait analysis), increased walking speed every session for 0.05 m/s | NR | NR |
| Chien et al., 2018 (6) | Progressively increasing perturbation intensity | NR | 80 perturbations in first week, 160 in week 2-8 | Preferred walking speed (10m gait analysis) | NR | NR |

Table A2. *Continued.*

| **Study** | **Training intensity and progression** | **Frequency of perturbations** | **Number of perturbations per training session** | **Treadmill belt speed (walking perturbations)** | **Gait event during perturbation (walking perturbations)** | **Perturbed leg (walking perturbations)** |
| --- | --- | --- | --- | --- | --- | --- |
| Dusane et al., 2021 (7) | Adjusted individually: intensity was increased when participant did not experience more than two falls on one given block | NR | 40 perturbations (divided into ten training blocks) | Preferred walking speed (10m gait analysis) | NR | NR |
| Faria et al., 2023 (8) | NR | Every 25-45 seconds | INT1 & INT2: 24 perturbations | Preferred walking speed (determined each session): increased and decreased to identify participants upper and lower boundaries, and using the mean as the final speed | NR | NR |
| Gassner et al. 2019 (9) | Adjusted individually: based on self-perceived exertion assessed by borg scale (6-20) with a target range of 12-15 and a Likert scale (1 "not difficult" to 7 "extremely difficult") with a target range of ≤5. Progressively increasing walking speed | NA | NA, divided into three blocks | Starting with 70% of self-selected overground walking speed, increasing walking speed | NA | NA |
| Gerards et al., 2023 (10) | Adjusted individually: 10-point scale of difficulty to maintain balance (6-9/10), progressively increasing perturbation intensity and walking speed | NR | NR | Ramp protocol starting at 0.5 m/s speed, gradually increasing until participant said "stop" | NR | NR |
| Gimmon et al., 2018 (11) | Adjusted individually: aim to provide a constant challenge, progressively increasing perturbation intensity | Every 20-30 seconds, randomized | NR | Preferred walking speed | Random in all phases of gait cycle | NR |

Table A2. *Continued.*

| **Study** | **Training intensity and progression** | **Frequency of perturbations** | **Number of perturbations per training sessions** | **Treadmill belt speed (walking perturbations)** | **Gait event during perturbation (walking perturbations)** | **Perturbed leg (walking perturbations)** |
| --- | --- | --- | --- | --- | --- | --- |
| Grabiner et al., 2012 (13) | Adjusted individually: increase in intensity when participant successfully recovered; progressively increasing perturbation intensity | NR | 15-30 perturbations | NA | NA | NA |
| Handelzalts et al., 2019 (14) | Adjusted individually: according to maximal ability (recovery without falling), progressively increasing perturbation intensity | NR | 66 (36 standing and 30 walking perturbations, divided into blocks of 12 perturbations) | NR | Mid-stance | NR |
| Hezel et al., 2023 (15) | Adjusted individually: based on the maximal ability assessed at baseline assessment and 5-point scale of subjectively perceived difficulty and anxiety (2-4/5), progressively increasing perturbation intensity | Every 10-20 seconds, randomized | 40 perturbations, divided into 5 blocks of 8 perturbations each (2-minute breaks in between) | Preferred walking speed; starting with 50% of overground walking speed (10 m gait analysis), increased and decreased to identify participants upper and lower boundaries, and using the mean as the final speed | Mid-stance | Both legs equally, randomized |
| Lanza et al., 2024 (16) | Adjusted individually: based on the step threshold in standing, progressively increasing perturbation intensity when 90% of trials were classified as successful recovery of balance | NR | 80 perturbations | NA | NA | NA |

Table A2. *Continued.*

| **Study** | **Training intensity and progression** | **Frequency of perturbations** | **Number of perturbations per training sessions** | **Treadmill belt speed (walking perturbations)** | **Gait event during perturbation (walking perturbations)** | **Perturbed leg (walking perturbations)** |
| --- | --- | --- | --- | --- | --- | --- |
| Lee et al., 2018 (17) | NR | NR | INT1: 24 perturbations, divided into 6 blocks of 4 perturbations each; INT2: 40 perturbations, divided into 10 blocks of 4 perturbations each | Preferred walking speed from four speed options (0.6, 0.8, 1.0, 1.2 m/s) | NR | NR |
| Liu et al., 2021 (18) | Adjusted individually: according to participants’ ability to tolerate intensity level | NR | 40 perturbations | Preferred walking speed from four speed options (0.6, 0.8, 1.0, 1.2 m/s) | NR | NR |
| Lurie et al., 2013 (19) | Progressively increasing perturbation intensity | NR | NR | Preferred walking speed | NR | NR |
| Lurie et al., 2020 (20) | Progressively increasing perturbation intensity | NR | NR | NR | NR | NR |
| Montana State University, 2021 (21) | Adjusted individually: according to maximal ability (recovery without falling) | Randomized frequency, not further defined | 24 perturbations | NA | NA | NA |
| Nachmani et al., 2021 (22) | Adjusted individually: based on step threshold in standing and adjusted to maximal ability (recovery without falling), progressively increasing perturbation intensity | Every 20-40 seconds, randomized | 35 perturbations | Preferred walking speed | Not defined and random by time | NR |

Table A2. *Continued.*

| **Study** | **Training intensity and progression** | **Frequency of perturbations** | **Number of perturbations per training sessions** | **Treadmill belt speed (walking perturbations)** | **Gait event during perturbation (walking perturbations)** | **Perturbed leg (walking perturbations)** |
| --- | --- | --- | --- | --- | --- | --- |
| Nørgaard et al., 2023 (23) | Adjusted individually: 5-point scale of subjectively perceived difficulty and anxiety (sum of both >4/10), progressively increasing perturbation intensity when score was ≤4, no fall occurred during the last 4 perturbations and the participant felt comfortable increasing the intensity | Every 10-50 steps, randomized | 40 perturbations, divided into 11 blocks of 2-4 perturbations each | Preferred walking speed; increased and decreased to identify participants upper and lower boundaries, and using the mean as the final speed | Treadmill belt deceleration at heel strike and belt acceleration at mid-swing of opposite leg | Both legs equally, randomized |
| Petrovic et al., 2024 (24) | Adjusted individually: adapted to participants' capacity determined by the therapist; progressively increasing perturbation intensity | NR | NR | Preferred walking speed | NR | NR |
| Protas et al., 2005 (25) | Progressively increasing perturbation intensity | NR | 25-35 perturbations | NA | NA | NA |
| Punt et al., 2019 (26) | Adjusted individually: RPE (no specification of the target range), progressively increasing perturbation intensity | Every 2-4 strides, randomized | NR | Preferred walking speed determined during first two minutes of each session; speed was frequently in- and decreased by researcher | Foot contact, mid-stance and foot off | Paretic and nonparetic limb |
| Rieger et al., 2020 (27) | Two different intensity levels | Every 10-60 seconds, randomized | 16 perturbations | Fixed at 1 m/s | Heel strike | Both legs randomized |
| Rieger et al., 2024 (28) | Adjusted individually: adjusted to participants’ ability not to grab the handrails to recover, progressively increasing perturbation intensity | Every 10-45 seconds, randomized | 37 perturbations (5 for measurement before training) | Preferred walking speed, speed was frequently increased by researcher | Foot contact | Both legs randomized |

Table A2. *Continued.*

| **Study** | **Training intensity and progression** | **Frequency of perturbations** | **Number of perturbations per training sessions** | **Treadmill belt speed (walking perturbations)** | **Gait event during perturbation (walking perturbations)** | **Perturbed leg (walking perturbations)** |
| --- | --- | --- | --- | --- | --- | --- |
| Shimada et al., 2004 (29) | Progressively increasing perturbation intensity (until max. of 100% reduction in speed) | NR | NR | 50/70% of max. speed varying between training phases; max. speed determined at the beginning of each session | NR | NR |
| US Department of Veterans Affairs, 2008 (30) | Increasing perturbation intensity | NR | NR | Preferred walking speed | NR | NR |
| Van Wouwe et al., 2021 (31) | Adjusted individually: based on the magnitude at which a step was needed to recover balance, progressively increasing perturbation intensity when the participants' step incidence was below 25% for the largest perturbation magnitude in the specific direction. | NR | 100 perturbations | NA | NA | NA |
| Virginia Polytechnic Institute and State University, 2022 (32) | NR | Randomized frequency, not further defined | NR | NA | NA | NA |
| Wang et al., 2022 (33) | 5 different intensity levels: increasing and then decreasing intensities | NR | 40 perturbations, divided into 11 blocks | NR | NR | NR |
| Whitten et al., 2023 (34) | NR | NR | Up to 35 perturbations | NR | NR | NR |

Table A2. *Continued.*

| **Study** | **Training intensity and progression** | **Frequency of perturbations** | **Number of perturbations per training sessions** | **Treadmill belt speed (walking perturbations)** | **Gait event during perturbation (walking perturbations)** | **Perturbed leg (walking perturbations)** |
| --- | --- | --- | --- | --- | --- | --- |
| Yang et al., 2021 (35) | NR | NR | NR | NR | NR | NR |
| Zhu et al., 2025 (36) | Adjusted individually: starting at 1.25 times of previously determined forward and backward limits of standing stability in baseline assessment, progressively increasing perturbation intensity | Randomized frequency, not further defined | 32 perturbations (divided into 3 training blocks) | Preferred walking speed (determined each session): increased and decreased to identify participants upper and lower boundaries, and using the mean as the final speed | Immediately after heel strike | Both legs randomized |
| Zieschang et al., 2024 (37) | NR | NR | NR | NR | NR | NR |

NA: not applicable (the training parameter is not applicable to the chosen perturbation modality); NR: not reported (no information about the training parameter is given in the study, although the Parameter applies to the perturbation modality); INT: Intervention; RPE: rate of perceived exertion.

*Note.* Only the main publication is listed. For additional related publications, see Table 2.

References

1. Allin LJ, Brolinson PG, Beach BM, Kim S, Nussbaum MA, Roberto KA, et al. Perturbation-based balance training targeting both slip- and trip-induced falls among older adults: A randomized controlled trial. BMC Geriatr. 2020;20:205. [https://doi.org/10.1186/s12877-020-01605-9](http://?)

2. Aviles J, Allin LJ, Alexander NB, Van Mullekom J, Nussbaum MA, Madigan ML. Comparison of Treadmill Trip-Like Training Versus Tai Chi to Improve Reactive Balance Among Independent Older Adult Residents of Senior Housing: A Pilot Controlled Trial. J Gerontol A Biol Sci Med Sci. 2019;74:1497-503. [https://doi.org/10.1093/gerona/glz018](http://?)

3. Bhatt T. Reactive Balance Training for Fall Prevention. 2018 [ClinicalTrials.gov identifier: NCT04205279]. Available from: [https://clinicaltrials.gov/study/NCT04205279?term=NCT04205279&rank=1](http://?).

4. Brüll L, Hezel N, Arampatzis A, Schwenk M. Comparing the Effects of Two Perturbation-Based Balance Training Paradigms in Fall-Prone Older Adults: A Randomized Controlled Trial. Gerontology. 2023;69:910-22. [https://doi.org/10.1159/000530167](http://?)

5. Cheng YS, Chien A, Lai DM, Lee YY, Cheng CH, Wang SF, et al. Perturbation-Based Balance Training in Postoperative Individuals With Degenerative Cervical Myelopathy.Front Bioeng Biotechnol. 2020;8:108. [https://doi.org/10.3389/fbioe.2020.00108](http://?)

6. Chien JE, Hsu WL. Effects of Dynamic Perturbation-Based Training on Balance Control of Community-Dwelling Older Adults. Sci Rep. 2018;8:17231. [https://doi.org/10.1038/s41598-018-35644-5](http://?)

7. Dusane S, Bhatt T. Effect of multisession progressive gait-slip training on fall-resisting skills of people with chronic stroke: Examining motor adaptation in reactive stability. 2021;11.894. [https://doi.org/10.3390/brainsci11070894](http://?)

8. Faria JO. Evaluation of the distribution of a Balance Training on the risk of falls and posture control in Elderly Fallers. 2023 [International Clinical Trials Registry Platform identifier: RBR-9dhx6kj]. Available from: [https://trialsearch.who.int/Trial2.aspx?TrialID=RBR-9dhx6kj](http://?).

9. Gaßner H, Steib S, Klamroth S, Pasluosta CF, Adler W, Eskofier BM, et al. Perturbation Treadmill Training Improves Clinical Characteristics of Gait and Balance in Parkinson's Disease. J Parkinsons Dis. 2019;9:413-26. [https://doi.org/10.3233/jpd-181534](http://?)

10. Gerards M, Marcellis R, Senden R, Poeze M, de Bie R, Meijer K, et al. The effect of perturbation-based balance training on balance control and fear of falling in older adults: a single-blind randomised controlled trial. BMC Geriatr. 2023;23:305. [https://doi.org/10.1186/s12877-023-03988-x](http://?)

11. Gimmon Y, Riemer R, Kurz I, Shapiro A, Debbi R, Melzer I. Perturbation exercises during treadmill walking improve pelvic and trunk motion in older adults—A randomized control trial. Arch Gerontol Geriatr. 2018;75:132-8. [https://doi.org/10.1016/j.archger.2017.12.004](http://?)

12. Shapiro A, Melzer I. Balance perturbation system to improve balance compensatory responses during walking in old persons. J Neuroeng Rehabil. 2010;7:32. [https://doi.org/10.1186/1743-0003-7-32](http://?)

13. Grabiner MD, Bareither ML, Gatts S, Marone J, Troy KL. Task-specific training reduces trip-related fall risk in women. Med Sci Sports Exerc. 2012;44:2410-4. [https://doi.org/10.1249/mss.0b013e318268c89f](http://?)

14. Handelzalts S, Kenner-Furman M, Gray G, Soroker N, Shani G, Melzer I. Effects of Perturbation-Based Balance Training in Subacute Persons With Stroke: A Randomized Controlled Trial. Neurorehabil Neural Repair. 2019;33:213-24. [https://doi.org/10.1177/1545968319829453](http://?)

15. Hezel N, Sloot LH, Wanner P, Becker C, Bauer JM, Steib S, et al. Feasibility, effectiveness and acceptability of two perturbation-based treadmill training protocols to improve reactive balance in fall-prone older adults (FEATURE): protocol for a pilot randomised controlled trial. BMJ Open. 2023;13:e073135. [https://doi.org/10.1136/bmjopen-2023-073135](http://?)

16. Lanza MB, Fujimoto M, Magder L, McCombe-Waller S, Rogers MW, Gray VL. Is lateral external perturbation training more beneficial for protective stepping responses than voluntary stepping training in stroke? A pilot randomized control study. J Neuroeng Rehabil. 2024;21:199. [https://doi.org/10.1186/s12984-024-01495-7](http://?)

17. Lee A, Bhatt T, Liu X, Wang Y, Pai YC. Can higher training practice dosage with treadmill slip-perturbation necessarily reduce risk of falls following overground slip? Gait Posture. 2018;61:387-92. [https://doi.org/10.1016/j.gaitpost.2018.01.037](http://?)

18. Liu X, Bhatt T, Wang Y, Wang S, Lee A, Pai YC. The retention of fall-resisting behavior derived from treadmill slip-perturbation training in community-dwelling older adults. Geroscience. 2021;43:913-26. [https://doi.org/10.1007/s11357-020-00270-5](http://?)

19. Lurie JD, Zagaria AB, Pidgeon DM, Forman JL, Spratt KF. Pilot comparative effectiveness study of surface perturbation treadmill training to prevent falls in older adults. BMC Geriatr. 2013;13:49. [https://doi.org/10.1186/1471-2318-13-49](http://?)

20. Lurie JD, Zagaria AB, Ellis L, Pidgeon D, Gill-Body KM, Burke C, et al. Surface Perturbation Training to Prevent Falls in Older Adults: A Highly Pragmatic, Randomized Controlled Trial. Phys Ther. 2020;100:1153-62. [https://doi.org/10.1093/ptj/pzaa023](http://?)

21. Montana State University. Learning From Falling: Perturbation-based Training to Prevent Falls in Older Adults. 2021 [ClinicalTrials.gov identifier: NCT04770103]. Available from: [https://clinicaltrials.gov/study/NCT04770103](http://?).

22. Nachmani H, Paran I, Salti M, Shelef I, Melzer I. Examining Different Motor Learning Paradigms for Improving Balance Recovery Abilities Among Older Adults, Random vs. Block Training—Study Protocol of a Randomized Non-inferiority Controlled Trial. Front Hum Neurosci. 2021;15:624492. [https://doi.org/10.3389/fnhum.2021.624492](http://?)

23. Nørgaard JE, Andersen S, Ryg J, Stevenson AJT, Andreasen J, Oliveira AS, et al. Effect of Treadmill Perturbation-Based Balance Training on Fall Rates in Community-Dwelling Older Adults: A Randomized Clinical Trial. JAMA Netw Open. 2023;6:e238422. [https://doi.org/10.1001/jamanetworkopen.2023.8422](http://?)

24. Petrovic A, Wirth R, Klimek C, Lueg G, Daubert D, Giehl C, et al. Impact of Reactive Balance Training on a Perturbation Treadmill on Physical Performance in Geriatric Patients:Results of a Single-Center, Assessor Blinded Randomized Controlled Trial. J Clin Med. 2024;13:5790. [https://doi.org/10.3390/jcm13195790](http://?)

25. Protas EJ, Mitchell K, Williams A, Qureshy H, Caroline K, Lai EC. Gait and step training to reduce falls in Parkinson's disease. NeuroRehabilitation. 2005;20:183-90. [https://doi.org/10.3233/NRE-2005-20305](http://?)

26. Punt M, Bruijn SM, Van De Port IG, De Rooij IJM, Wittink H, Van Dieën JH. Does a perturbation-based gait intervention enhance gait stability in fall-prone stroke survivors? A pilot study. J Appl Biomech. 2019;35:173-81. [https://doi.org/10.1123/jab.2017-0282](http://?)

27. Rieger MM, Papegaaij S, Pijnappels M, Steenbrink F, van Dieën JH. Transfer and retention effects of gait training with anterior-posterior perturbations to postural responses after medio-lateral gait perturbations in older adults. Clin Biomech. 2020;75:104988. [https://doi.org/10.1016/j.clinbiomech.2020.104988](http://?)

28. Rieger MM, Papegaaij S, Steenbrink F, van Dieën JH, Pijnappels M. Effects of Perturbation-Based Treadmill Training on Balance Performance, Daily Life Gait, and Falls in Older Adults: REACT Randomized Controlled Trial. Phys Ther. 2024;104:pzad136. [https://doi.org/10.1093/ptj/pzad136](http://?)

29. Shimada H, Obuchi S, Furuna T, Suzuki T. New intervention program for preventing falls among frail elderly people: The effects of perturbed walking exercise using a bilateral separated treadmill. Am J Phys Med Rehabil. 2004;83:493-9. [https://doi.org/10.1097/01.phm.0000130025.54168.91](http://?)

30. US Department of Veteran Affairs. Fall Prevention Program for Older Adults. 2008 [ClinicalTrials.gov identifier: NCT00714051]. Available from: [https://clinicaltrials.gov/study/NCT00714051](http://?).

31. Van Wouwe T, Afschrift M, Dalle S, Van Roie E, Koppo K, De Groote F. Adaptations in Reactive Balance Strategies in Healthy Older Adults After a 3-Week Perturbation Training Program and After a 12-Week Resistance Training Program. Front Sports Act Living. 2021;3:714555. [https://doi.org/10.3389/fspor.2021.714555](http://?)

32. Virginia Polytechnic Institute and State University. Effects of Task-Specific Step Training on Reactive Balance. 2022 [ClinicalTrials.gov identifier: NCT05734443]. Available from: [https://clinicaltrials.gov/study/NCT05734443](http://?).

33. Wang Y, Wang S, Liu X, Lee A, Pai YC, Bhatt T. Can a single session of treadmill-based slip training reduce daily life falls in community-dwelling older adults? A randomized controlled trial. Aging Clin Exp Res. 2022;34:1593-602. [https://doi.org/10.1007/s40520-022-02090-3](http://?)

34. Whitten J, Graham D, Grocke M, O’Leary B, Riley J, Tarabochia D, et al. The feasibility and acceptability of perturbation balance training in rural communities: a mixed methods study. Innovation in Aging. 2023;7:598.

35. Yang F. Perturbation Training Reduces Falls in People With AD (STAD) 2021 [ClinicalTrials.gov identifier: NCT05205980]. Available from: [https://clinicaltrials.gov/study/NCT05205980](http://?).

36. Zhu RT-L, Schulte FA, Singh NB, Zong-Hao C, Awai Easthope C, Ravi DK, et al. Effects of Single-Session Perturbation-Based Balance Training with Progressive Intensities on Resilience and Dynamic Gait Stability in Healthy Older Adults. Front Bioeng Biotechnol. 2025:13:1642158. [https://doi.org/10.3389/fbioe.2025.1642158](http://?)

37. Zieschang T. Perturbation-Based Treadmill Training to Prevent Unrecovered Falls in Geriatric Patients (TRAIL) 2024 [ClinicalTrials.gov identifier: NCT06652828]. Available from: [https://clinicaltrials.gov/study/NCT06652828](http://?).
