# Supplementary material for: Perturbation-based balance training on treadmills for falls prevention in older adults: a review of training protocols and reporting recommendations (ProRePBT)
Source: BMC Geriatr. 2026 Feb 14;26:300. doi: 10.1186/s12877-026-07124-3 (PMC12958537; doi:10.1186/s12877-026-07124-3)
Supplement: Supplementary file 3 — Supplementary Material 3. [file 12877_2026_7124_MOESM3_ESM.docx]

**Additional file 3.**

Table A3. *Reported justifications for general information, training period and number of training sessions, training session duration, type and predictability of perturbations.*

| **Study** | **General Information** | **Number of training sessions and training period** | **Duration of total training session; duration of perturbation training** | **Type of perturbation** | **Predictability of perturbations** |
| --- | --- | --- | --- | --- | --- |
| Allin et al., 2020 (1) | General statement about trip training justified based on previous studies (2, 3) | NR | NR | NR | General statement about randomization in order „to prevent participants from anticipating“ justified based on authors’ assumptions |
| Aviles et al., 2019 (2) | NR | NR | NR | NR | General statement about randomization in order to reduce anticipation and maintain variability justified based on authors’ assumptions |
| Bhatt et al., 2018 (4) | NR | NR | NR | NR | NR |
| Brüll et al., 2023 (5) | General statement about training protocols being based on recommendations for implementing PBT in clinical practice in a previous review (6) | NR | NR | General statement that the high perturbation variety was based on recommendations in a previous review (6) | General statement about randomization in order to reduce „the predictability of the upcoming perturbation, minimizing proactive adjustments to focus on reactive performance“ justified based on authors’ assumptions |
| Cheng et al., 2020 (7) | NR | NR | NR | NR | Unpredictability of perturbations justified based on previous study (8) |
| Chien et al., 2018 (8) | NR | NR | NR | NR | NR |
| Dusane et al., 2021 (9) | NR | NR | NR | NR | NR |

Table A3. *Continued.*

| **Study** | **General Information** | **Number of training sessions and training period** | **Duration of total training session; duration of perturbation training** | **Type of perturbation** | **Predictability of perturbations** |
| --- | --- | --- | --- | --- | --- |
| Faria et al., 2023 (10) | NR | NR | NR | NR | Unannounced perturbations justified based on previous study (11) |
| Gassner et al., 2019 (12) | General statement that measurement protocol is justified based on previous study (13); general statement about treadmill familiarization justified based on previous study (14); general statement about restricted feedback to reduce influence of feedback while training justified based on authors’ assumptions; general statement about giving minimal instructions during familiarization for safety reasons based on authors’ assumptions | NR | NR | NR | NR |
| Gerards et al., 2023 (15) | NR | NR | Duration of perturbation training justified based on previous studies (11, 16-21) | Types of perturbation justified based on recommendation in a previous review (6) | NR |
| Gimmon et al., 2018 (22) | NR | NR | NR | NR | General statement about random order of perturbations because of ecological validity justified based on authors’ assumptions |

Table A3. *Continued.*

| **Study** | **General Information** | **Number of training sessions and training period** | **Duration of total training session; duration of perturbation training** | **Type of perturbation** | **Predictability of perturbations** |
| --- | --- | --- | --- | --- | --- |
| Grabiner et al., 2012 (3) | NR | NR | NR | NR | General statement about randomization in order „to reduce anticipation“ justified based on authors’ assumptions |
| Handelzalts et al., 2019 (23) | General statement about specificity of training justified based on previous guidelines (24) | NR | NR | NR | NR |
| Hezel et al., 2023 (25) | General statement that PBT protocol is based on a previous study protocol (26) | NR | Duration of total training justified based on feasibility aspects | NR | NR |
| Lanza et al., 2024 (27) | General statement that training parameters are justified based on previous study (28) | NR | NR | NR | NR |
| Lee et al., 2018 (29) | General statement that PBT training sessions are structured based on the principle of block-and-mixed practice (30) | NR | NR | NR | NR |
| Liu et al., 2021 (31) | NR | NR | NR | NR | General statement about unannounced perturbations “in order to mimic real-life slips” justified based on authors’ assumptions |
| Lurie et al., 2013 (16) | NR | NR | NR | NR | NR |

Table A3. *Continued.*

| **Study** | **General Information** | **Number of training sessions and training period** | **Duration of total training session; duration of perturbation training** | **Type of perturbation** | **Predictability of perturbations** |
| --- | --- | --- | --- | --- | --- |
| Lurie et al., 2020 (32) | NR | NR | Duration of training session justified based on authors' experience of feasibility in a previous pilot study (16) | NR | NR |
| Montana State University, 2021 (33) | General statement that training protocol considering principles of motor learning is justified based on a previous study (34) | NR | NR | NR | NR |
| Nachmani et al., 2021 (35) | General statement that the training protocol is based on principles of physical training and exercise prescriptions (6, 30, 36-38); general statement that focus on cognitive understanding of training was for improving self-confidence justified based on authors’ assumptions | NR | NR | NR | Random order of perturbation types justified based on motor learning principles (39, 40) |
| Nørgaard et al., 2023 (41) | Treadmill familiarization in the first session justified based on recommendations in previous studies (42-44) | Booster training session justified based on previous study (30) | NR | NR | Predictable perturbations in initial sessions justified based on previous studies (43, 44); random order of perturbations in following sessions justified based on recommendations in previous review and study (45, 46) |

Table A3. *Continued.*

| **Study** | **General Information** | **Number of training sessions and training period** | **Duration of total training session; duration of perturbation training** | **Type of perturbation** | **Predictability of perturbations** |
| --- | --- | --- | --- | --- | --- |
| Petrovic et al., 2024 (47) | General statement that no standard training protocol was possible because of the multimorbid population justified based on authors’ assumptions | NR | NR | NR | NR |
| Protas et al., 2005 (17) | NR | NR | NR | NR | NR |
| Punt et al., 2019 (48) | General statement that intervention design is justified based on principles for motor learning (49) | Training period justified based on recommendations from previous study (50) | NR | NR | NR |
| Rieger et al., 2020 (51) | General statement about treadmill familiarization justified based on recommendation of previous study (42); general statement about detaching handrails to not use handrails justified based on previous study (52) | NR | NR | Types of perturbation justified based on previous study (53) | NR |
| Rieger et al., 2024 (54) | NR | NR | NR | Multiple types of perturbation justified based on recommendations of previous systematic review and study (44, 45) | Randomization of perturbation types justified based on recommendations of previous systematic review and study (44, 45) |
| Shimada et al., 2004 (20) | NR | NR | NR | NR | NR |

Table A3. *Continued.*

| **Study** | **General Information** | **Number of training sessions and training period** | **Duration of total training session; duration of perturbation training** | **Type of perturbation** | **Predictability of perturbations** |
| --- | --- | --- | --- | --- | --- |
| US Department of Veterans Affairs, 2008 (55) | NR | NR | NR | NR | NR |
| Van Wouwe et al., 2021 (56) | NR | NR | NR | NR | Randomization of perturbation types to “minimize anticipatory postural adjustment” justified based on authors’ assumption |
| Virginia Polytechnic Institute and State University, 2022 (57) | NR | NR | NR | NR | NR |
| Wang et al., 2022 (58) | PBT protocol justified based on the procedures of previous study (59) | NR | NR | NR | NR |
| Whitten et al., 2023 (60) | NR | NR | NR | NR | NR |
| Yang et al., 2021 (61) | NR | NR | NR | NR | NR |
| Zhu et al., 2025 (62) | NR | NR | NR | NR | NR |
| Zieschang et al., 2024 (63) | NR | NR | NR | NR | NR |

NA: not applicable (the training parameter is not applicable to the chosen perturbation modality); NR: not reported (no information about the training parameter is given in the study, although the Parameter applies to the perturbation modality).

*Note.* Only the main publication is listed. For additional related publications, see Table 2.

Table A4. *Reported justifications for training intensity and progression, perturbation frequency, number of perturbations, treadmill belt speed, gait event during perturbation and perturbed leg.*

| **Study** | **Training intensity and progression** | **Frequency of perturbations** | **Number of perturbations per training session** | **Treadmill belt speed (walking perturbations)** | **Gait event during perturbation (walking perturbations)** | **Perturbed leg (walking perturbations)** |
| --- | --- | --- | --- | --- | --- | --- |
| Allin et al., 2020 (1) | NR | NR | NR | NA | NA | NA |
| Aviles et al., 2019 (2) | Perturbation intensity was chosen based on previous evidence but modified to maintain variability justified based on authors’ experience (6) | NR | NR | NA | NA | NA |
| Bhatt et al., 2018 (4) | NR | NR | NR | NR | NR | NR |
| Brüll et al., 2023 (5) | Progression of intensity justified based on authors’ experience in a previous study (no reference); 5-point scale for progression justified based on previous study (44) | Frequency justified based on authors’ experience in previous study | NR | NR | Gait phase justified based on recommendations in previous review (6) | Perturbed leg justified based on recommendations in previous review (6) |
| Cheng et al., 2020 (7) | NR | NR | Number of perturbations per perturbation type justified based on previous study (53) | NR | NR | NR |
| Chien et al., 2018 (8) | NR | NR | NR | Treadmill belt speed justified based on previous study (64) | NR | NR |
| Dusane et al., 2021 (9) | NR | NR | NR | NR | NR | NR |
| Faria et al., 2023 (10) | NR | NR | NR | Treadmill belt speed justified based on previous studies (65, 66) | NR | NR |

Table A4. *Continued.*

| **Study** | **Training intensity and progression** | **Frequency of perturbations** | **Number of perturbations per training session** | **Treadmill belt speed (walking perturbations)** | **Gait event during perturbation (walking perturbations)** | **Perturbed leg (walking perturbations)** |
| --- | --- | --- | --- | --- | --- | --- |
| Gassner et al., 2019 (12) | Progression of intensity adaptation justified based on previous study (67) | NA | NA | NR | NA | NA |
| Gerards et al., 2023 (15) | Perturbation intensity was chosen based on previous evidence but was modified based on authors’ experience (6) | NR | NR | NR | NR | NR |
| Gimmon et al., 2018 (22) | NR | NR | NR | NR | General statement about perturbations in all phases of the gait cycle because of ecological validity justified based on authors’ assumptions | NR |
| Grabiner et al., 2012 (3) | NR | NR | NR | NA | NA | NA |
| Handelzalts et al., 2019 (23) | NR | NR | NR | NR | NR | NR |
| Hezel et al., 2023 (25) | Intensity and 5-point scale for progression based on previous study protocol (26, 44, 68) | NR | NR | Treadmill belt speed justified based on previous study protocol (26) | NR | NR |
| Lanza et al., 2024 (27) | NR | NR | NR | NA | NA | NA |

Table A4. *Continued.*

| **Study** | **Training intensity and progression** | **Frequency of perturbations** | **Number of perturbations per training session** | **Treadmill belt speed (walking perturbations)** | **Gait event during perturbation (walking perturbations)** | **Perturbed leg (walking perturbations)** |
| --- | --- | --- | --- | --- | --- | --- |
| Lee et al., 2018 (29) | NR | NR | Group with 24 perturbations justified based on the procedures of previous study (69) Group with 40 perturbations justified based on authors’ assumptions of potential enhanced effects of more repetitions | NR | NR | NR |
| Liu et al., 2021 (31) | Progression based on previous study (59) | NR | Number of perturbations justified based on previous study (70) | NR | NR | NR |
| Lurie et al., 2013 (16) | NR | NR | NR | NR | NR | NR |
| Lurie et al., 2020 (32) | NR | NR | NR | NR | NR | NR |
| Montana State University, 2021 (33) | NR | NR | NR | NA | NA | NA |
| Nachmani et al., 2021 (35) | General statement that progression was based on principles of physical training (40) | NR | NR | NR | NR | NR |

Table A4. *Continued.*

| **Study** | **Training intensity and progression** | **Frequency of perturbations** | **Number of perturbations per training session** | **Treadmill belt speed (walking perturbations)** | **Gait event during perturbation (walking perturbations)** | **Perturbed leg (walking perturbations)** |
| --- | --- | --- | --- | --- | --- | --- |
| Nørgaard et al., 2023 (41) | General statement that training protocol and progression are justified based on previous studies (70, 71) | NR | Number of perturbations justified based on recommendations in previous study (29) | Treadmill belt speed justified based on recommendations in previous studies (72, 73) | NR | NR |
| Petrovic et al., 2024 (47) | NR | NR | NR | NR | NR | NR |
| Protas et al., 2005 (17) | NR | NR | NR | NA | NA | NA |
| Punt et al., 2019 (48) | NR | Frequency of perturbations justified based on authors’ experience | NR | NR | NR | NR |
| Rieger et al., 2020 (51) | Perturbation intensity justified based on previous study (53) | NR | Number of perturbations justified based on recommendations in previous studies (74, 75) | NR | NR | NR |
| Rieger et al., 2024 (54) | NR | Random time intervals between perturbations justified based on recommendations of previous systematic review and study (44, 45) | NR | Increase of treadmill belt speed justified based on recommendations in previous study (72) | NR | Randomization of perturbed leg justified based on recommendations of previous systematic review and study (44, 45) |

Table A4. *Continued.*

| **Study** | **Training intensity and progression** | **Frequency of perturbations** | **Number of perturbations per training session** | **Treadmill belt speed (walking perturbations)** | **Gait event during perturbation (walking perturbations)** | **Perturbed leg (walking perturbations)** |
| --- | --- | --- | --- | --- | --- | --- |
| Shimada et al., 2004 (20) | NR | NR | NR | NR | NR | NR |
| US Department of Veterans Affairs, 2008 (55) | NR | NR | NR | NR | NR | NR |
| Van Wouwe et al., 2021 (56) | NR | NR | NR | NA | NA | NA |
| Virginia Polytechnic Institute and State University, 2022 (57) | NR | NR | NR | NA | NA | NA |
| Wang et al., 2022 (58) | NR | NR | NR | NR | NR | NR |
| Whitten et al., 2023 (60) | NR | NR | NR | NR | NR | NR |
| Yang et al., 2021 (61) | NR | NR | NR | NR | NR | NR |
| Zhu et al., 2025 (62) | NR | NR | NR | NR | NR | NR |
| Zieschang et al., 2024 (63) | NR | NR | NR | NR | NR | NR |

NA: not applicable (the training parameter is not applicable to the chosen perturbation modality); NR: not reported (no information about the training parameter is given in the study, although the Parameter applies to the perturbation modality).

*Note.* Only the main publication is listed. For additional related publications, see Table 2.

**References**

1. Allin LJ, Brolinson PG, Beach BM, Kim S, Nussbaum MA, Roberto KA, et al. Perturbation-based balance training targeting both slip- And trip-induced falls among older adults: A randomized controlled trial. BMC Geriatr. 2020;20:205. [https://doi.org/10.1186/s12877-020-01605-9](http://?)

2. Aviles J, Allin LJ, Alexander NB, Van Mullekom J, Nussbaum MA, Madigan ML. Comparison of Treadmill Trip-Like Training Versus Tai Chi to Improve Reactive Balance Among Independent Older Adult Residents of Senior Housing: A Pilot Controlled Trial. J Gerontol A Biol Sci Med Sci. 2019;74:1497-503. [https://doi.org/10.1093/gerona/glz018](http://?)

3. Grabiner MD, Bareither ML, Gatts S, Marone J, Troy KL. Task-specific training reduces trip-related fall risk in women. Med Sci Sports Exerc. 2012;44:2410-4. [https://doi.org/10.1249/mss.0b013e318268c89f](http://?)

4. Bhatt T. Reactive Balance Training for Fall Prevention. 2018 [ClinicalTrials.gov identifier: NCT04205279]. Available from: [https://clinicaltrials.gov/study/NCT04205279?term=NCT04205279&rank=1](http://?).

5. Brüll L, Hezel N, Arampatzis A, Schwenk M. Comparing the Effects of Two Perturbation-Based Balance Training Paradigms in Fall-Prone Older Adults: A Randomized Controlled Trial. Gerontology. 2023;69:910-22. [https://doi.org/10.1159/000530167](http://?)

6. Gerards MHG, McCrum C, Mansfield A, Meijer K. Perturbation-based balance training for falls reduction among older adults: Current evidence and implications for clinical practice. Geriatr Gerontol Int. 2017;17:2294-303. [https://doi.org/10.1111/ggi.13082](http://?)

7. Cheng YS, Chien A, Lai DM, Lee YY, Cheng CH, Wang SF, et al. Perturbation-Based Balance Training in Postoperative Individuals With Degenerative Cervical Myelopathy. Front Bioeng Biotechnol. 2020;8:108. [https://doi.org/10.3389/fbioe.2020.00108](http://?)

8. Chien JE, Hsu WL. Effects of Dynamic Perturbation-Based Training on Balance Control of Community-Dwelling Older Adults. Sci Rep. 2018;8:17231. [https://doi.org/10.1038/s41598-018-35644-5](http://?)

9. Dusane S, Bhatt T. Effect of multisession progressive gait-slip training on fall-resisting skills of people with chronic stroke: Examining motor adaptation in reactive stability. Brain Sci. 2021;11:894. [https://doi.org/10.3390/brainsci11070894](http://?)

10. Faria JO. Evaluation of the distribution of a Balance Training on the risk of falls and posture control in Elderly Fallers. 2023 [International Clinical Trials Registry Platform identifier: RBR-9dhx6kj]. Available from: [https://trialsearch.who.int/Trial2.aspx?TrialID=RBR-9dhx6kj](http://?).

11. Pai YC, Bhatt T, Yang F, Wang E. Perturbation Training Can Reduce Community-Dwelling Older Adults’ Annual Fall Risk: A Randomized Controlled Trial. J Gerontol A Biol Sci Med Sci. 2014;69:1586-94. [https://doi.org/10.1093/gerona/glu087](http://?)

12. Gaßner H, Steib S, Klamroth S, Pasluosta CF, Adler W, Eskofier BM, et al. Perturbation Treadmill Training Improves Clinical Characteristics of Gait and Balance in Parkinson's Disease. J Parkinsons Dis. 2019;9:413-26. [https://doi.org/10.3233/jpd-181534](http://?)

13. Bello O, Sanchez JA, Fernandez-del-Olmo M. Treadmill walking in Parkinson's disease patients: Adaptation and generalization effect. Mov Disord. 2008;23:1243-9. [https://doi.org/10.1002/mds.22069](http://?)

14. Klamroth S, Steib S, Gaßner H, Goßler J, Winkler J, Eskofier B, et al. Immediate effects of perturbation treadmill training on gait and postural control in patients with Parkinson's disease. Gait Posture. 2016;50:102-8. [https://doi.org/10.1016/j.gaitpost.2016.08.020](http://?)

15. Gerards M, Marcellis R, Senden R, Poeze M, de Bie R, Meijer K, et al. The effect of perturbation-based balance training on balance control and fear of falling in older adults: a single-blind randomised controlled trial. BMC Geriatr. 2023;23:305. [https://doi.org/10.1186/s12877-023-03988-x](http://?)

16. Lurie JD, Zagaria AB, Pidgeon DM, Forman JL, Spratt KF. Pilot comparative effectiveness study of surface perturbation treadmill training to prevent falls in older adults. BMC Geriatr. 2013;16:49. [https://doi.org/10.1186/1471-2318-13-49](http://?)

17. Protas EJ, Mitchell K, Williams A, Qureshy H, Caroline K, Lai EC. Gait and step training to reduce falls in Parkinson's disease. NeuroRehabilitation. 2005;20:183-90. [https://doi.org/10.3233/NRE-2005-20305](http://?)

18. Rosenblatt NJ, Marone J, Grabiner MD. Preventing trip-related falls by community-dwelling adults: a prospective study. J Am Geriatr Soc. 2013;61:1629-31. [https://doi.org/10.1111/jgs.12428](http://?)

19. Shen X, Mak MKY. Technology-assisted balance and gait training reduces falls in patients with Parkinson's disease: a randomized controlled trial with 12-month follow-up. Neurorehabil Neural Repair. 2015;29:103-11. [https://doi.org/10.1177/1545968314537559](http://?)

20. Shimada H, Obuchi S, Furuna T, Suzuki T. New intervention program for preventing falls among frail elderly people: The effects of perturbed walking exercise using a bilateral separated treadmill. Am J Phys Med Rehabil. 2004;83:493-9. [https://doi.org/10.1097/01.phm.0000130025.54168.91](http://?)

21. Smania N, Corato E, Tinazzi M, Stanzani C, Fiaschi A, Girardi P, et al. Effect of balance training on postural instability in patients with idiopathic Parkinson's disease. Neurorehabil Neural Repair. 2010;24:826-34. [https://doi.org/10.1177/1545968310376057](http://?)

22. Gimmon Y, Riemer R, Kurz I, Shapiro A, Debbi R, Melzer I. Perturbation exercises during treadmill walking improve pelvic and trunk motion in older adults—A randomized control trial. Arch Gerontol Geriatr. 2018;75:132-8. [https://doi.org/10.1016/j.archger.2017.12.004](http://?)

23. Handelzalts S, Kenner-Furman M, Gray G, Soroker N, Shani G, Melzer I. Effects of Perturbation-Based Balance Training in Subacute Persons With Stroke: A Randomized Controlled Trial. Neurorehabil Neural Repair. 2019;33:213-24. [https://doi.org/10.1177/1545968319829453](http://?)

24. Febinger L. American College of Sports Medicine. Guidelines for Exercises Testing and Prescription. 1990;4th edition.

25. Hezel N, Sloot LH, Wanner P, Becker C, Bauer JM, Steib S, et al. Feasibility, effectiveness and acceptability of two perturbation-based treadmill training protocols to improve reactive balance in fall-prone older adults (FEATURE): protocol for a pilot randomised controlled trial. BMJ Open. 2023;13:73135. [https://doi.org/10.1136/bmjopen-2023-073135](http://?)

26. Nørgaard JE, Andersen S, Ryg J, Stevenson AJT, Andreasen J, Danielsen MB, et al. Effects of treadmill slip and trip perturbation-based balance training on falls in community-dwelling older adults (STABILITY): study protocol for a randomized controlled trial. BMJ Open. 2022;12:e052492. [https://doi.org/10.1136/bmjopen-2021-052492](http://?)

27. Lanza MB, Fujimoto M, Magder L, McCombe-Waller S, Rogers MW, Gray VL. Is lateral external perturbation training more beneficial for protective stepping responses than voluntary stepping training in stroke? A pilot randomized control study. J Neuroeng Rehabil. 2024;21:199. [https://doi.org/10.1186/s12984-024-01495-7](http://?)

28. Gray VL, Westlake KP. The Feasibility of Lateral Externally-Induced Perturbation Training in Fall Prevention after Stroke. Int J Cerebrovasc Dis Stroke. 2024;7:174. [https://doi.org/10.29011/2688-8734.100174](http://?)

29. Lee A, Bhatt T, Liu X, Wang Y, Pai YC. Can higher training practice dosage with treadmill slip-perturbation necessarily reduce risk of falls following overground slip? Gait Posture. 2018;61:387-92. [https://doi.org/10.1016/j.gaitpost.2018.01.037](http://?)

30. Bhatt T, Yang F, Pai Y-C. Learning to resist gait-slip falls: long-term retention in community-dwelling older adults. Arch Phys Med Rehabil. 2012;93:557-64. [https://doi.org/10.1016/j.apmr.2011.10.027](http://?)

31. Liu X, Bhatt T, Wang Y, Wang S, Lee A, Pai YC. The retention of fall-resisting behavior derived from treadmill slip-perturbation training in community-dwelling older adults. Geroscience. 2021;43:913-26. [https://doi.org/10.1007/s11357-020-00270-5](http://?)

32. Lurie JD, Zagaria AB, Ellis L, Pidgeon D, Gill-Body KM, Burke C, et al. Surface Perturbation Training to Prevent Falls in Older Adults: A Highly Pragmatic, Randomized Controlled Trial. Phys Ther. 2020;100:1153-62. [https://doi.org/10.1093/ptj/pzaa023](http://?)

33. Montana State University. Learning From Falling: Perturbation-based Training to Prevent Falls in Older Adults. 2021 [ClinicalTrials.gov identifier: NCT04770103]. Available from: [https://clinicaltrials.gov/study/NCT04770103](http://?).

34. Mansfield A, Aqui A, Danells CJ, Knorr S, Centen A, Depaul VG, et al. Does perturbation-based balance training prevent falls among individuals with chronic stroke? A randomised controlled trial. BMJ Open. 2018;8:e021510. [https://doi.org/10.1136/bmjopen-2018-021510](http://?)

35. Nachmani H, Paran I, Salti M, Shelef I, Melzer I. Examining Different Motor Learning Paradigms for Improving Balance Recovery Abilities Among Older Adults, Random vs. Block Training—Study Protocol of a Randomized Non-inferiority Controlled Trial. Front Hum Neurosci. 2021;15:624492. [https://doi.org/10.3389/fnhum.2021.624492](http://?)

36. Mansfield A, Wong JS, Bryce J, Knorr S, Patterson KK. Does Perturbation-Based Balance Training Prevent Falls? Systematic Review and Meta-Analysis of Preliminary Randomized Controlled Trials. Phys Ther. 2015;95:700-9. [https://doi.org/10.2522/ptj.20140090](http://?)

37. Okubo Y, Schoene D, Lord SR. Step training improves reaction time, gait and balance and reduces falls in older people: a systematic review and meta-analysis. Br J Sports Med. 2017;51:586-93. [https://doi.org/10.1136/bjsports-2015-095452](http://?)

38. Sherrington C, Fairhall NJ, Wallbank GK, Tiedemann A, Michaleff ZA, Howard K, et al. Exercise for preventing falls in older people living in the community. Cochrane Database Syst Rev. 2019;1:CD012424. [https://doi.org/10.1002/14651858.cd012424.pub2](http://?)

39. Dick MB, Hsieh S, Dick-Muehlke C, Davis DS, Cotman CW. The variability of practice hypothesis in motor learning: does it apply to Alzheimer's disease? Brain Cogn. 2000;44:470-89. [https://doi.org/10.1006/brcg.2000.1206](http://?)

40. Drowatzky KL, Drowatzky JN. Physical training programs for the elderly. 1999. p. 53, 2-62.

41. Nørgaard JE, Andersen S, Ryg J, Stevenson AJT, Andreasen J, Oliveira AS, et al. Effect of Treadmill Perturbation-Based Balance Training on Fall Rates in Community-Dwelling Older Adults: A Randomized Clinical Trial. JAMA Netw Open. 2023;6:e238422. [https://doi.org/10.1001/jamanetworkopen.2023.8422](http://?)

42. Meyer C, Killeen T, Easthope CS, Curt A, Bolliger M, Linnebank M, et al. Familiarization with treadmill walking: How much is enough? Sci Rep. 2019;9:5232. [https://doi.org/10.1038/s41598-019-41721-0](http://?)

43. Okubo Y, Brodie MA, Sturnieks DL, Hicks C, Lord SR. A pilot study of reactive balance training using trips and slips with increasing unpredictability in young and older adults: Biomechanical mechanisms, falls and clinical feasibility. Clin Biomech. 2019;67:171-9. [https://doi.org/10.1016/j.clinbiomech.2019.05.016](http://?)

44. Okubo Y, Sturnieks DL, Brodie MA, Duran L, Lord SR. Effect of Reactive Balance Training Involving Repeated Slips and Trips on Balance Recovery Among Older Adults: A Blinded Randomized Controlled Trial. J Gerontol A Biol Sci Med Sci. 2019;74:1489-96. [https://doi.org/10.1093/gerona/glz021](http://?)

45. McCrum C, Gerards MHG, Karamanidis K, Zijlstra W, Meijer K. A systematic review of gait perturbation paradigms for improving reactive stepping responses and falls risk among healthy older adults. Eur Rev Aging Phys Act. 2017;14:3. [https://doi.org/10.1186/s11556-017-0173-7](http://?)

46. Takazono PS, Ribeiro de Souza C, Ávila de Oliveira J, Coelho DB, Teixeira LA. High contextual interference in perturbation-based balance training leads to persistent and generalizable stability gains of compensatory limb movements. Exp Brain Res. 2020;238:1249-63. [https://doi.org/10.1007/s00221-020-05806-x](http://?)

47. Petrovic A, Wirth R, Klimek C, Lueg G, Daubert D, Giehl C, et al. Impact of Reactive Balance Training on a Perturbation Treadmill on Physical Performance in Geriatric Patients:Results of a Single-Center, Assessor Blinded Randomized Controlled Trial. J Clin Med. 2024;13:5790. [https://doi.org/10.3390/jcm13195790](http://?)

48. Punt M, Bruijn SM, Van De Port IG, De Rooij IJM, Wittink H, Van Dieën JH. Does a perturbation-based gait intervention enhance gait stability in fall-prone stroke survivors? A pilot study. J Appl Biomech. 2019;35:173-81. [https://doi.org/10.1123/jab.2017-0282](http://?)

49. Guadagnoll MA, Lee TD. Challenge Point: A Framework for Conceptualizing the Effects of Various Practice Conditions in Motor Learning. J Mot Behav. 2004;36:212-24. [https://doi.org/10.3200/jmbr.36.2.212-224](http://?)

50. Pohl M, Mehrholz J, Ritschel C, Rückriem S. Speed-dependent treadmill training in ambulatory hemiparetic stroke patients: a randomized controlled trial. Stroke. 2002;33:553-8. [https://doi.org/10.1161/hs0202.102365](http://?)

51. Rieger MM, Papegaaij S, Pijnappels M, Steenbrink F, van Dieën JH. Transfer and retention effects of gait training with anterior-posterior perturbations to postural responses after medio-lateral gait perturbations in older adults. Clin Biomech. 2020;75:104988. [https://doi.org/10.1016/j.clinbiomech.2020.104988](http://?)

52. Buurke TJW, Lamoth CJC, Van Der Woude LHV, Den Otter R. Handrail holding during treadmill walking reduces locomotor learning in able-bodied persons. IEEE Trans Neural Syst Rehabil Eng. 2019;27:1753-9. [https://doi.org/10.1109/tnsre.2019.2935242](http://?)

53. Roeles S, Rowe PJ, Bruijn SM, Childs CR, Tarfali GD, Steenbrink F, et al. Gait stability in response to platform, belt, and sensory perturbations in young and older adults. Med Biol Eng Comput. 2018;56:2325-35. [https://doi.org/10.1007/s11517-018-1855-7](http://?)

54. Rieger MM, Papegaaij S, Steenbrink F, van Dieën JH, Pijnappels M. Effects of Perturbation-Based Treadmill Training on Balance Performance, Daily Life Gait, and Falls in Older Adults: REACT Randomized Controlled Trial. Phys Ther. 2024;104:pzad136. [https://doi.org/10.1093/ptj/pzad136](http://?)

55. US Department of Veteran Affairs. Fall Prevention Program for Older Adults. 2008 [ClinicalTrials.gov identifier: NCT00714051]. Available from: [https://clinicaltrials.gov/study/NCT00714051](http://?).

56. Van Wouwe T, Afschrift M, Dalle S, Van Roie E, Koppo K, De Groote F. Adaptations in Reactive Balance Strategies in Healthy Older Adults After a 3-Week Perturbation Training Program and After a 12-Week Resistance Training Program. Front Sports Act Living. 2021;3:714555. [https://doi.org/10.3389/fspor.2021.714555](http://?)

57. Virginia Polytechnic Institute and State University. Effects of Task-Specific Step Training on Reactive Balance. 2022 [ClinicalTrials.gov identifier: NCT05734443]. Available from: [https://clinicaltrials.gov/study/NCT05734443](http://?).

58. Wang Y, Wang S, Liu X, Lee A, Pai YC, Bhatt T. Can a single session of treadmill-based slip training reduce daily life falls in community-dwelling older adults? A randomized controlled trial. Aging Clin Exp Res. 2022;34:1593-602. [https://doi.org/10.1007/s40520-022-02090-3](http://?)

59. Wang Y, Bhatt T, Liu X, Wang S, Lee A, Wang E, et al. Can treadmill-slip perturbation training reduce immediate risk of over-ground-slip induced fall among community-dwelling older adults? J Biomech. 2019;84:58-66. [https://doi.org/10.1016/j.jbiomech.2018.12.017](http://?)

60. Whitten J, Graham D, Grocke M, O’Leary B, Riley J, Tarabochia D. The feasibility and acceptability of perturbation balance training in rural communities: a mixed methods study. Innovation in Aging. 2023;7:598.

61. Yang F. Perturbation Training Reduces Falls in People With AD (STAD). 2021 [ClinicalTrials.gov identifier: NCT05205980]. Available from: [https://clinicaltrials.gov/study/NCT05205980](http://?).

62. Zhu RT-L, Schulte FA, Singh NB, Zong-Hao C, Awai Easthope C, Ravi DK. Effects of Single-Session Perturbation-Based Balance Training with Progressive Intensities on Resilience and Dynamic Gait Stability in Healthy Older Adults. Front Bioeng Biotechnol. 2025;13:1642158. [https://doi.org/10.3389/fbioe.2025.1642158](http://?)

63. Zieschang T. Perturbation-Based Treadmill Training to Prevent Unrecovered Falls in Geriatric Patients (TRAIL). 2024 [ClinicalTrials.gov identifier: NCT06652828]. Available from: [https://clinicaltrials.gov/study/NCT06652828](http://?).

64. Kozlowska K, Latka M, West BJ. Asymmetry of short-term control of spatio-temporal gait parameters during treadmill walking. Sci Rep. 2017;7:44349. [https://doi.org/10.1038/srep44349](http://?)

65. Dal U, Erdogan T, Resitoglu B, Beydagi H. Determination of preferred walking speed on treadmill may lead to high oxygen cost on treadmill walking. Gait Posture. 2010;31(3):366-9. [https://doi.org/10.1016/j.gaitpost.2010.01.006](http://?)

66. Jordan K, Challis JH, Newell KM. Walking speed influences on gait cycle variability. Gait Posture. 2007;26(1):128-34. [https://doi.org/10.1016/j.gaitpost.2006.08.010](http://?)

67. Steib S, Klamroth S, Gassner H, Pasluosta C, Eskofier B, Winkler J, et al. Perturbation During Treadmill Training Improves Dynamic Balance and Gait in Parkinson's Disease: A Single-Blind Randomized Controlled Pilot Trial. Neurorehabil Neural Repair. 2017;31(8):758-68. [https://doi.org/10.1177/1545968317721976](http://?)

68. Song PYH, Sturnieks DL, Davis MK, Lord SR, Okubo Y. Perturbation-Based Balance Training Using Repeated Trips on a Walkway vs. Belt Accelerations on a Treadmill: A Cross-Over Randomised Controlled Trial in Community-Dwelling Older Adults. Front Sports Act Living. 2021;3:702320. [https://doi.org/10.3389/fspor.2021.702320](http://?)

69. Pai YC, Yang F, Bhatt T, Wang E. Learning from laboratory-induced falling: long-term motor retention among older adults. Age. 2014;36:1367-76. [https://doi.org/10.1007/s11357-014-9640-5](http://?)

70. Lee A, Bhatt T, Liu X, Wang Y, Wang S, Pai YCC. Can treadmill slip-perturbation training reduce longer-term fall risk upon overground slip exposure? J Appl Biomech. 2020;36:298-306. [https://doi.org/10.1123/jab.2019-0211](http://?)

71. Wang Y, Wang S, Lee A, Pai YC, Bhatt T. Treadmill-gait slip training in community-dwelling older adults: mechanisms of immediate adaptation for a progressive ascending-mixed-intensity protocol. Exp Brain Res. 2019;237:2305-17. [https://doi.org/10.1007/s00221-019-05582-3](http://?)

72. Kang HG, Dingwell JB. Effects of walking speed, strength and range of motion on gait stability in healthy older adults. J Biomech. 2008;41:2899-905. [https://doi.org/10.1016/j.jbiomech.2008.08.002](http://?)

73. Row Lazzarini BS, Kataras TJ. Treadmill walking is not equivalent to overground walking for the study of walking smoothness and rhythmicity in older adults. Gait Posture. 2016;46:42-6. [https://doi.org/10.1016/j.gaitpost.2016.02.012](http://?)

74. Yang F, Cereceres P, Qiao M. Treadmill-based gait-slip training with reduced training volume could still prevent slip-related falls. Gait Posture. 2018;66:160-5. [https://doi.org/10.1016/j.gaitpost.2018.08.029](http://?)

75. Pai YC, Bhatt T, Wang E, Espy D, Pavol MJ. Inoculation against falls: rapid adaptation by young and older adults to slips during daily activities. Arch Phys Med Rehabil. 2010;91(3):452-9. [https://doi.org/10.1016/j.apmr.2009.10.032](http://?)
